# Retargeting azithromycin analogues to have dual-modality antimalarial activity

**DOI:** 10.1186/s12915-020-00859-4

**Published:** 2020-09-29

**Authors:** Amy L. Burns, Brad E. Sleebs, Ghizal Siddiqui, Amanda E. De Paoli, Dovile Anderson, Benjamin Liffner, Richard Harvey, James G. Beeson, Darren J. Creek, Christopher D. Goodman, Geoffrey I. McFadden, Danny W. Wilson

**Affiliations:** 1 Research Centre for Infectious Diseases, School of Biological Sciences, The University of Adelaide, Adelaide, 5005 Australia; 2 Walter and Eliza Hall Institute of Medical Research, Melbourne, Victoria 3050 Australia; 3 Department of Medical Biology, University of Melbourne, Melbourne, Victoria 3050 Australia; 4 Monash Institute of Pharmaceutical Sciences, Monash University, Melbourne, Victoria 3052 Australia; 5 Burnet Institute, Melbourne, Victoria 3004 Australia; 6 Department of Medicine, University of Melbourne, Melbourne, Australia; 7 Central Clinical School and Department of Microbiology, Monash University, Melbourne, Australia; 8 School of Biosciences, University of Melbourne, Melbourne, Victoria 3010 Australia

**Keywords:** *Plasmodium*, Malaria, Antimalarial, Macrolide

## Abstract

**Background:**

Resistance to front-line antimalarials (artemisinin combination therapies) is spreading, and development of new drug treatment strategies to rapidly kill *Plasmodium* spp. malaria parasites is urgently needed. Azithromycin is a clinically used macrolide antibiotic proposed as a partner drug for combination therapy in malaria, which has also been tested as monotherapy. However, its slow-killing ‘delayed-death’ activity against the parasite’s apicoplast organelle and suboptimal activity as monotherapy limit its application as a potential malaria treatment. Here, we explore a panel of azithromycin analogues and demonstrate that chemical modifications can be used to greatly improve the speed and potency of antimalarial action.

**Results:**

Investigation of 84 azithromycin analogues revealed nanomolar quick-killing potency directed against the very earliest stage of parasite development within red blood cells. Indeed, the best analogue exhibited 1600-fold higher potency than azithromycin with less than 48 hrs treatment in vitro. Analogues were effective against zoonotic *Plasmodium knowlesi* malaria parasites and against both multi-drug and artemisinin-resistant *Plasmodium falciparum* lines. Metabolomic profiles of azithromycin analogue-treated parasites suggested activity in the parasite food vacuole and mitochondria were disrupted. Moreover, unlike the food vacuole-targeting drug chloroquine, azithromycin and analogues were active across blood-stage development, including merozoite invasion, suggesting that these macrolides have a multi-factorial mechanism of quick-killing activity. The positioning of functional groups added to azithromycin and its quick-killing analogues altered their activity against bacterial-like ribosomes but had minimal change on ‘quick-killing’ activity. Apicoplast minus parasites remained susceptible to both azithromycin and its analogues, further demonstrating that quick-killing is independent of apicoplast-targeting, delayed-death activity.

**Conclusion:**

We show that azithromycin and analogues can rapidly kill malaria parasite asexual blood stages via a fast action mechanism. Development of azithromycin and analogues as antimalarials offers the possibility of targeting parasites through both a quick-killing and delayed-death mechanism of action in a single, multifactorial chemotype.

## Background

Malaria is a mosquito-borne disease caused by protozoan parasites of the genus *Plasmodium*. In 2017, there were ~ 219 million cases of malaria that resulted in ~ 435,000 deaths [1, 2], with most deaths as the result of *Plasmodium falciparum* infection in children under 5 years of age within sub-Saharan Africa. Current control strategies include use of insecticide treated bed-nets and indoor residual spraying, which target mosquito transmission, chemoprophylaxis in high-risk groups, and artemisinin combination therapies (ACTs) to both cure patients and limit their transmission. Widespread use of these control measures has resulted in significant decreases in malaria mortality over the past two decades [1, 2]. However, there is growing concern that artemisinin-resistant *P. falciparum* parasites may spread from the Greater Mekong sub-region and Eastern India, where they have previously been identified, and will lead to the loss of our most effective drug treatments [3–6]. Furthermore, there is also substantial resistance to some of the current partner drugs used in ACTs, most notably piperaquine and mefloquine [7]. Therefore, new antimalarials with novel mechanisms of action that rapidly clear blood-stage parasites are urgently needed [8, 9].

Clinically used macrolide antibiotics, in particular azithromycin, have been proposed as partner drugs for ACTs [10, 11]. Macrolide antibiotics have been shown to target the malaria parasite’s remnant plastid (apicoplast), which has a bacterium-like ribosomal complex essential for protein translation and organelle biogenesis [12–14]. The apicoplast is essential for synthesis of isopentenyl pyrophosphate (IPP) precursors required for protein prenylation, ubiquinone biosynthesis and dolichols required for N-glycosylation and production of GPI anchors (reviewed in [15] and [16]). Indeed, IPP synthesis is the sole essential function of the apicoplast in blood stages, but apicoplast biogenesis and housekeeping activity is essential for IPP production, making the apicoplast ribosome an attractive antimalarial target [13, 14, 17]. *P. falciparum* parasites treated with clinically relevant (nanomolar) concentrations of macrolide antibiotics exhibit a ‘delayed-death’ phenotype in which parasite growth is arrested during the second replication cycle after treatment (~ 4 days post-treatment) [13, 14].

Azithromycin exhibits three favourable properties as an antimalarial: a half-life > 50 hrs making it suitable for infrequent dosing [18], good in vivo safety profile [19] and high potency against *P. falciparum* in vitro [20, 21]. Azithromycin also shows efficacy as a prophylactic [22] (reviewed in [23]), improved clinical outcomes in combination with pyrimethamine during intermittent preventative treatment for malaria in pregnancy (IPTp) trials [24] and led to a significant decrease in *P. falciparum* infections following mass drug administrations of azithromycin monotherapy for trachoma infection [25]. Evidence also suggests that azithromycin inhibits the development of mosquito transmissible parasites and liver stages in rodent models [22, 26, 27]. However, when azithromycin was trialled for treatment of clinical malaria, it exhibited sub-optimal activity as a monotherapy and was generally less effective than the similarly acting antibiotic clindamycin when used in combination with other antimalarials [28]. Crucially, the delayed-death activity of azithromycin has limited its use as a treatment for clinical disease. Currently, azithromycin is not used as a first-line treatment for malaria because of these considerations.

We previously demonstrated that azithromycin can also cause rapid parasite death when tested at higher concentrations (IC_50_ ~ 10 μM) [27, 29]. Most strikingly, azithromycin can rapidly inhibit *P. falciparum* merozoite invasion of RBCs at these higher concentrations. In addition, azithromycin kills parasites within one intracellular blood-stage lifecycle (from immediately post-merozoite invasion to final schizont maturation at 48 hrs, in-cycle) at a similar IC_50_ as the drug’s invasion inhibitory activity. Testing of a small panel of azithromycin analogues showed that these ‘quick-killing’ IC_50_s could be enhanced through chemical modification. Importantly, parasites selected for resistance to azithromycin’s delayed-death activity (120 hr post-invasion) remained susceptible to both invasion-inhibition and intracellular parasite quick-killing activities (invasion, in-cycle and 72 hr inhibition), indicating that azithromycin has a secondary, apicoplast-independent, mechanism of action [27, 29]. Therefore, chemical modification of azithromycin presents a unique opportunity to develop a dual-acting antimalarial with two independent mechanisms of action that combines both quick-killing (for rapid clearance of clinical infection) and delayed-death activities, providing an element of resistance proofing and improving longer-term protection from recrudescence or reinfection.

In this study, we screened 84 azithromycin analogues and defined their efficacy against different stages of the blood-stage lifecycle. A high proportion of analogues exhibited improved quick-killing activity over azithromycin against both *P. falciparum* and *P. knowlesi*, a model for *P. vivax* and human pathogen of developing importance in Southeast Asia [30], and were equally effective against parasites containing or lacking an apicoplast. The analogues acted rapidly at inhibitory concentrations with only short treatment times required to kill parasites throughout blood-stage development. Given the established safety profile, long-half life, low cost of manufacture, and previous evaluation in ACTs, the re-development of azithromycin-like compounds into an antimalarial with dual mechanisms of action provides a novel strategy to develop new antimalarials.

## Results

### Azithromycin analogues show improvement in quick-killing activity against *P. falciparum*

**Fig. 1.**
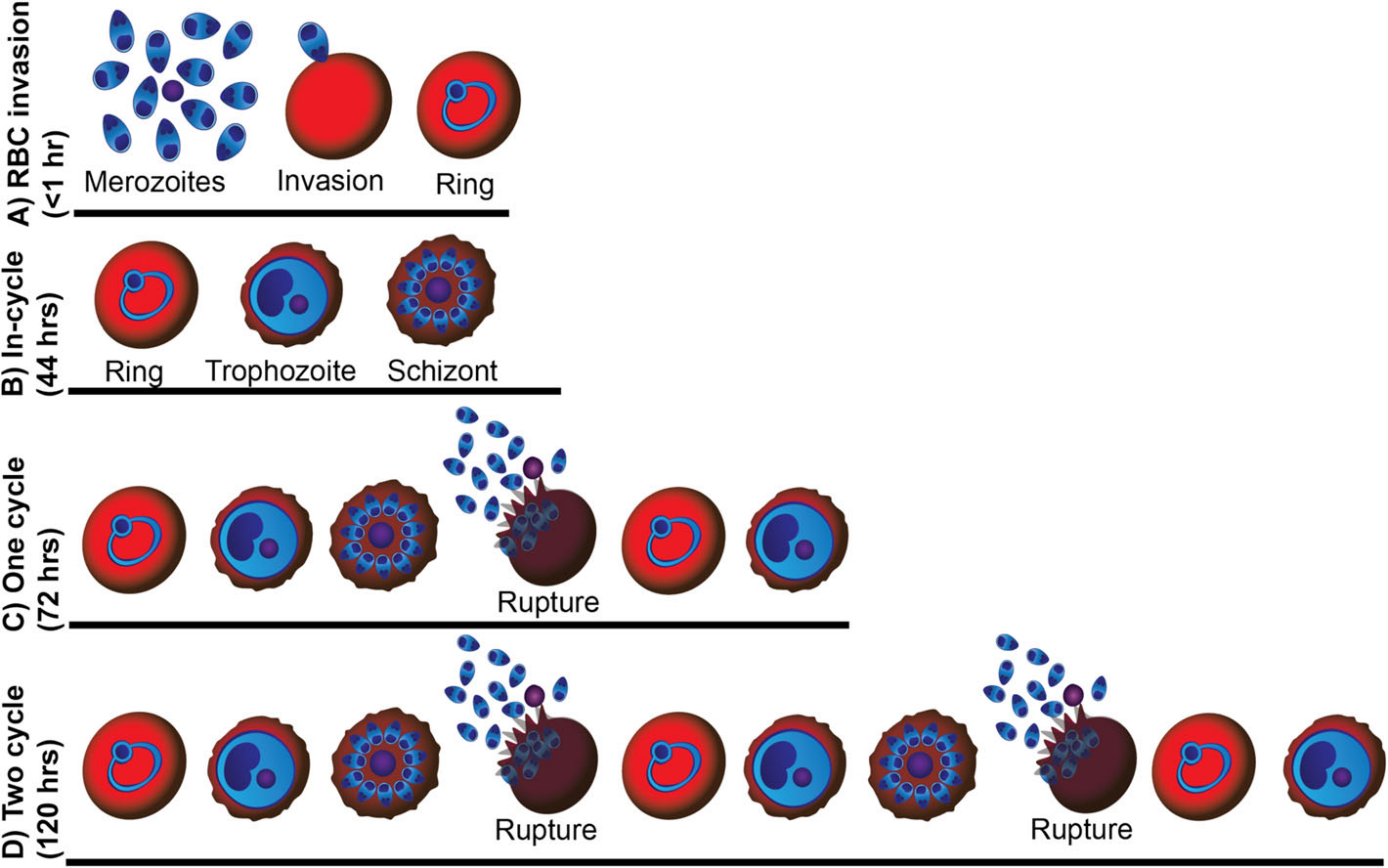
Schematic of drug treatment regimens outlining the times of treatment and stage/time of parasitaemia measurement for assays used in this study. **a** Merozoite invasion of RBCs: Merozoites were drug treated prior to addition of RBCs. RBC invasion was measured at early ring stages (< 1 hr rings). **b** In-cycle: highly synchronous, early ring-stage parasites (0–4 hrs post-invasion) were treated with drug, with the resulting growth inhibition analysed at schizont stage (44 hrs post-invasion for *P. falciparum* and 26 hrs for *P. knowlesi*). **c** One cycle (0–72 hrs): highly synchronous, early ring-stage parasites (0–4 hrs post-invasion) were drug-treated and the resulting growth inhibition was measured after ~ 72 hrs of growth, post one cycle of re-invasion, at schizont stages. **d** 2 cycle (delayed death); highly synchronous, early ring-stage parasites (0–4 hrs post-invasion) were drug-treated and allowed to grow for 92 hrs before washing drug with fresh media (post second invasion cycle). Growth inhibition was assessed approximately 30 hrs later, at schizont stages (0–120 hrs post-invasion for *P. falciparum* and 0–92 hrs for *P. knowlesi*)

**Fig. 2.**
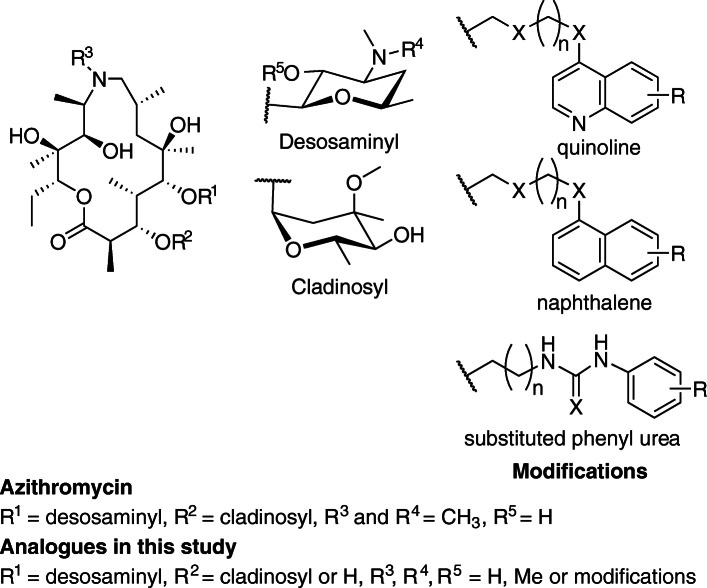
Structure of azithromycin and analogues. Outline of the structure of the parent molecule azithromycin, structural side-chains and sites of attachment of functional groups (R^1–5^) for compounds shown in Table 1. Structure of functional groups added is listed in Table 1

We characterised the activity of 84 azithromycin analogues across the malaria parasites asexual blood-stage development in fine detail, including their activity against early ring stages. The IC_50_ values for 72 hr growth-inhibition assays (drug treatment assays represented in Fig. 1; 1 cycle assay Fig. 1c) and their toxicity against mammalian cells for analogues presented in this study have been published previously [31–35]. Here, we tested for quick-killing activity using 44 hr in-cycle assays, wherein 10 μM of drug was added to early ring-stage D10-PfPHG parasites within a few hours of invasion and parasite development quantified at late schizont stage with no exposure of invading merozoites to the drug. This initial screen identified 65 of 84 analogues that inhibited growth by > 30% (Fig. 1b, Additional file 1: Tables S1a-c). The in-cycle IC_50_ values for these 65 analogues were determined (Additional file 1: Tables S1a-c) with all but two analogues showing improved potency over azithromycin (azithromycin IC_50_ with 44 hr in-cycle treatment, 11.3 μM) with the most potent compound exhibiting a 1615-fold lower IC_50_ than azithromycin (GSK-66 IC_50_ 0.007 μM). Notably, 39 analogues showed > 10-fold improvement over azithromycin (IC_50_ < 1 μM), with 16 exhibiting a > 55-fold improvement (IC_50_ < 0.2 μM). Summary inhibitory assay data and structure for 19 of the most potent analogues featuring different added functional groups is available in Table 1 and Fig. 2. Published cytotoxicity data against mammalian cells is available for 13 of the most potent analogues [31, 33–35] with the IC_50_ against the HepG2 cell line ranging between 3 and 83 μM and the selectivity index (SI; IC_50_ against HepG2/44 hr D10-PfPHG IC_50_ from this study) ranging between 15 to 415 fold. Eleven of these analogues had a SI > 50, indicating low mammalian cell toxicity.

**Table 1:** In vitro efficacy of antimalarials and azithromycin analogues against *Plasmodium* spp. parasites

cmpnd	R^3^ class	R^4^ class	R^5^ class	In-cycle (44 hr) growth D10-*Pf*PHG IC_50_ (μM^a^, ±SEM)	In-cycle (44 hr) growth DD2 IC_50_ (μM^b^, ±SEM)	Invasion inhibition D10-*Pf*PHG IC_50_ (μM^c^, ±SEM)	In-cycle (24 hr) growth *Pk*YH1 IC_50_ (μM^d^, ±SEM)
AZR	Me	Me	H	11.31 *(0.49)*	15.6 *(2.1)*	10 *(1.4)*	16 *(1.8)*
CQ				0.052 *(0.006)*	0.31 *(0.31)*	ND	0.017 *(0.005)*
QN				0.39 *(0.07)*	ND	ND	ND
DHA				0.0008 *(0.0001)*	ND	ND	0.0024 *(0.001)*
1	Chloroquinoline	Me	H	0.019 *(0.004)*	0.082 *(0.02)*	ND	0.2 *(0.005)*
56	Me	Chloroquinoline	H	0.011 *(0.002)*	0.093 *(0.02)*	3.2 *(0.39)*	0.031 *(0.008)*
59	Me	Chloroquinoline	H	0.073 *(0.02)*	0.049 *(0.005)*	ND	ND
66	Me	Me	Chloroquinoline	0.007 *(0.001)*	0.043 *(0.002)*	ND	0.012 *(0.002)*
69	Me	Me	Chloroquinoline	0.031 *(0.004)*	ND	ND	ND
70	Me	Me	Chloroquinoline	0.05 *(0.006)*	ND	ND	ND
72	Me	Me	Chloroquinoline	0.27 *(0.01)*	0.065 *(0.004)*	1.7 *(0.02)*	0.15 *(0.06)*
8	Quinoline	Me	H	0.41 *(0.02)*	0.52 *(0.1)*	4.4 *(1.2)*	0.15 *(0.01)*
10	Quinoline	Me	H	0.48 *(0.04)*	0.748 *(0.1)*	ND	0.1 *(0.005)*
58	Me	Quinoline	H	0.048 *(0.004)*	0.056 *(0.01)*	ND	0.071 *(0.013)*
71	Me	Me	Quinoline	0.053 *(0.005)*	0.16 *(0.02)*	ND	0.041 *(0.005)*
73	Me	Me	Quinoline	0.31 *(0.02)*	0.48 *(0.2)*	ND	0.248 *(0.07)*
3	Naphthalene	Me	H	0.183 *(0.02)*	0.32 *(0.07)*	1.8 *(0.5)*	0.095 *(0.02)*
4	Naphthalene	Me	H	0.19 *(0.01)*	ND	2.0 *(0.2)*	ND
15	Naphthalene	Me	H	0.67 *(0.07)*	0.4 *(0.1)*	3.6 *(0.4)*	0.32 *(0.12)*
5	Substituted phenyl thiourea	Me	H	0.2 *(0.01)*	0.4 *(0.05)*	1.61 *(0.02)*	0.082 *(0.02)*
6	Substituted phenyl thiourea	Me	H	0.28 *(0.05)*	0.27 *(0.07)*	ND	0.16 *(0.03)*
9	Substituted phenyl thiourea	Me	H	0.44 *(0.07)*	0.24 *(0.04)*	ND	0.016 *(0.005)*
17	Substituted phenyl thiourea	Me	H	0.7 *(0.05)*	0.54 *(0.06)*	ND	0.36 *(0.01)*

^a^ Drug treatment of intracellular growth, from rings to late schizonts, with no rupture cycle for D10-*Pf*PHG (*P. falciparum,* 0–44 hrs). Data represents the mean of 3 or more experiments

^b^ Drug treatment of intracellular growth, from rings to late schizonts, with no rupture cycle for DD2 (*P. falciparum,* 0–44 hrs). Data represents the mean of 3 or more experiments

^c^ Drug treatment of D10-*Pf*PHG merozoites prior to addition of RBCs. Parasitemia was measured by flow cytometry ~ 30 min post invasion. Data represents the mean of 2 (for compounds 4 and 5) or 3 experiments

^d^ Drug treatment of intracellular growth, from rings to late schizonts, with no rupture cycle for *P. knowlesi* YH1 (*P. knowlesi,* 0–24 h). Data represents the mean of 2 or more experiments

The analogues with the low nanomolar 44 hr in-cycle activity often featured quinoline or chloroquinoline modifications (Table 1, Fig. 2, Additional file 1: Tables S1a-c). However, there were exceptions including a number of phenyl-substituted analogues (GSK-5, GSK-6, GSK-9, GSK-11, GSK-14, GSK-16, GSK-17, GSK-19) and naphthalene-substituted analogues (GSK-3, GSK-4, GSK-15, GSK-18), which all displayed IC_50_ values < 1 μM. There was no structural difference between the most potent analogues and the analogues with activity > 1 μM that could explain the observed activity discrepancy. Consistently, chloroquinoline analogues (GSK-1, GSK-2, GSK-56 and GSK-66) were more potent than their respective unsubstituted quinoline counterparts (GSK-7, GSK-10, GSK-58 and GSK-71). Analogues GSK-6 and GSK-9 with thiourea aryl substitution displayed comparable potency (IC_50_ 0.2 and 0.44 μM) to naphthalene analogues GSK-3 and GSK-4 (IC_50_ 0.18 and 0.19 μM). However, a large number of analogues supporting thiourea and urea aryl substitutions were significantly less active, with no clear distinction between the activity and substitution pattern on the aryl ring of thiourea- or urea-substituted analogues.

Analogues with aliphatic substitution on the urea or thiourea (GSK-31, GSK-35, GSK-38, GSK-45, GSK-47, GSK-51) generally had reduced activity compared to analogues with pendant aryl moieties (Table 1, Fig. 2, Additional file 1: Tables S1a-c), suggesting the aryl substituent was important for modulating potency. Consistent with this observation, analogues that did not terminate with an aromatic substituent and were only decorated with small aliphatic functionality (analogues GSK-34, GSK-44, GSK-46, GSK-50, GSK-52, GSK-53, GSK-54, GSK-55, GSK-62, GSK-64, GSK-83, GSK-84) were either weakly active (> 3 μM) or inactive. These data suggested that the type of functionality and the length of the carbon-chain linking the aromatic group to the macrolactone was not important for activity. However, analogues GSK-56, GSK-57, GSK-58, GSK-66, GSK-67 and GSK-71, with short 3-carbon linkers between the macrolactone and the quinoline group, were amongst the most potent. Overall, there was no consistent trend between the type of functionality and the length of the carbon-chain linking the aromatic group to the macrolactone.

The position of the pendant quinoline or aromatic system attached to the macrolactone—either N6-, O-desosaminyl or N-desosaminyl—did not affect the in-cycle 44 hr activity of analogues (Table 1, Fig. 2, Additional file 1: Tables S1a-c). For example, analogues with the same quinoline functionality, GSK-1, GSK-56 and GSK-66 attached to either N6-, N-desosaminyl or O-desosaminyl positions, displayed similar IC_50_ values between 7 and 19 nM. This trend was observed amongst other analogues for which there were matched pairs. The cladinosyl group did not affect 44 hr in-cycle activity, for example respective analogues with the cladinosyl group, GSK-1, GSK-10, GSK-56 and GSK-66, possessed similar activity compared to analogues without the cladinosyl group, GSK-67, GSK-7 and GSK-57. This observation is consistent with our previous findings on the azalide structure activity relationship [29].

### Azithromycin analogues show improved activity against merozoite RBC invasion

We previously showed that azithromycin and analogues inhibit merozoite invasion, with merozoites found to contact and briefly deform the RBC membrane, and then detach when examined in the presence of azithromycin [29]. We investigated whether the 39 analogues that had an in-cycle (44 hr) IC_50_ < 1 μM could inhibit merozoite invasion at a concentration of 1 μM and identified eight analogues that inhibited invasion by > 20% at 1 μM (Fig. 1a, Table 1, Fig. 2, Additional file 1: Tables S1a-c). The invasion inhibitory IC_50_ for seven of these analogues with sufficient available sample were determined; there was a 2- to 6-fold reduction in the invasion inhibitory IC_50_ over azithromycin (range GSK-8 4.4 μM to GSK-5 1.6 μM) (Table 1, Additional file 2: Figure S1). Importantly, azithromycin analogues with improved in-cycle activity also had improved potency against merozoite invasion, confirming previous observations that both invasion and in-cycle quick-killing activities can be improved with a single chemical modification [29]. We next tested whether azithromycin analogue invasion inhibitory activity was directed against the merozoite by treating purified merozoites with 10 μM of GSK-72 (invasion inhibitory IC_50_ 1.7 μM), followed by washing drug off the merozoites, and then mixing merozoites with RBCs (Additional file 3 Figure S2). GSK-72-treated merozoites were stopped from invading RBCs after washing off the drug, suggesting that the invasion inhibitory activity of azithromycin analogues is irreversible and directed towards the merozoite.

### Quick-killing activity is independent of apicoplast targeting

We previously showed that quick-killing activity is maintained against delayed-death-resistant parasites [29], suggesting that quick-killing occurs through a mechanism of action independent of the apicoplast. However, the fact that the apicoplast and apicoplast-ribosome were still present in these drug-treated parasites left open the possibility that quick-killing activity could still be linked to the apicoplast [36]. To confirm quick-killing is completely independent of the apicoplast, we generated apicoplast minus (*Pf*PHG^apicoplast-null^) parasites through prolonged treatment with azithromycin and then rescued with media supplementation with the isoprenoid precursor, isopentenyl pyrophosphate (IPP) [17, 36]. *Pf*PHG^apicoplast-null^ parasites showed a complete loss of sensitivity to azithromycin in 120 hr delayed-death assays, confirming that the apicoplast had been removed (Additional file 4: Figure S3a) [17, 36]. In contrast, there was no difference in growth inhibition for the *Pf*PHG^apicoplast-null^ and *Pf*PHG^wildtype^ parasites when treated with azithromycin (Additional file 4: Figure S3b) and 15 lead analogues at the in-cycle D10-*Pf*PHG^wildtype^ IC_90_ concentration for 44 hrs (Additional file 4: Figure S3c; Additional file 1: Table S1a-b). These data confirm that quick-killing activity is independent of the apicoplast, indicating that there is a secondary mechanism of action for azithromycin and analogues.

### Azithromycin is a rapid and irreversible inhibitor across blood-stage parasite growth

**Fig. 3.**
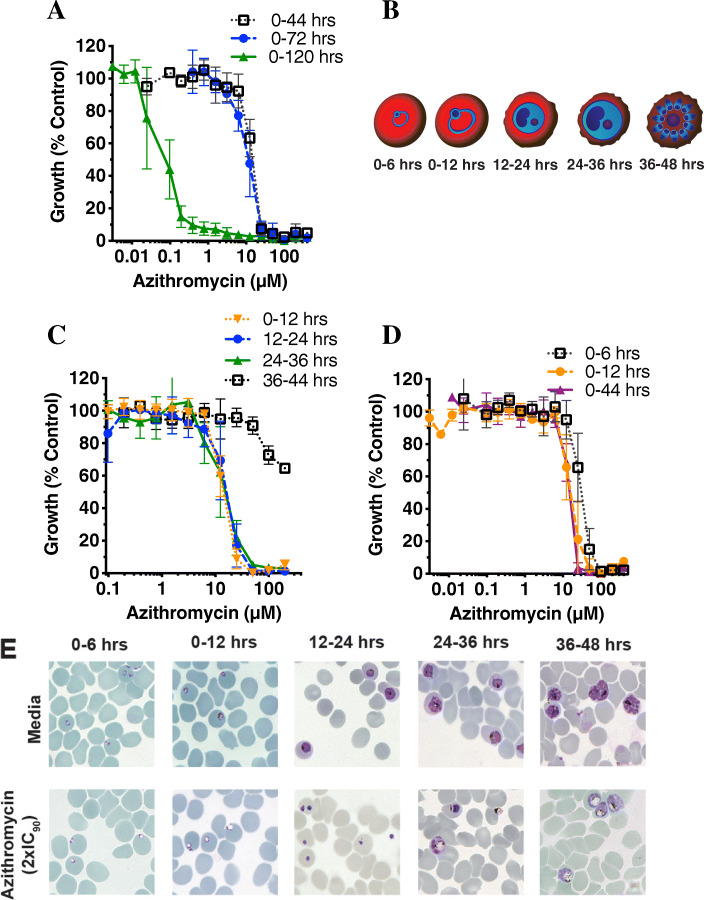
Azithromycin has broad activity against blood-stage parasites. **a** Early ring-stage *P. falciparum* parasites (0–4 hrs post-invasion) were treated with doubling dilutions of azithromycin and inhibition of growth measured for in-cycle (44 hr, IC_50_, 11 μM), 1-cycle (72 hr, IC_50_, 14 μM) and 2-cycle (delayed death, 120 hr, IC_50_, 0.07 μM) assays (44 hr vs 72 hr, P=NS; 120 hr vs 44 hr *P* < 0.0001; 120 hr vs 72 hr P < 0.0001). **b** Schematic of drug washout treatment scheme to assess azithromycin’s quick-killing stage of activity. Early ring-stage parasites (0–4 hrs post-invasion) were aliquoted to a 96-well plate and doubling dilutions of azithromycin added between 0-12 hrs, 12–24 hrs, 24–36 hrs and 36–44 hrs post invasion prior to drug removal by washing with fresh media. **c** Growth inhibition of azithromycin across 0–12 hrs, 12–24 hrs, 24–36 hrs and 36–44 hrs post invasion prior to drug removal by washing with fresh media. There was no significance between treatment times for 0–12 hrs, 12–24 hrs, 24–36 hrs, but there was for 0–12 hrs vs 36–44 hrs (*P* = 0.005), 12–24 hrs vs 36–44 hrs (*P* = 0.01) and 24–36 hrs vs 36–44 hrs (*P* = 0.01). **d** Growth inhibition of azithromycin with very early ring-stage treatment across 0–6 hrs and 0–12 hrs post-invasion compared to a full in-cycle (0–44 hr) treatment. Treatments showed significant difference (*P* < 0.0001) with the exception of 0–12 hrs vs 0–44 hrs (*P* = 0.19). For all growth curves, parasitemia was measured at 44 hrs post invasion at schizont stage via flow cytometry. Data represents the means of 3 or more experiments expressed as a percentage of non-inhibitory control and error bars represent ± SEM. Dose response IC_50_s compared using extra sum of squares *F*-test. Repeat measure data is available in Additional file 15 Supporting Value Data. **e** Representative Giemsa-stained thin blood smears showing the growth phenotypes seen for non-inhibitory media controls (top panels) and in the presence of 2× IC_90_ concentration of azithromycin (bottom panels) across different stages of intraerythrocytic blood-stage development (0–6 hrs, 0–12 hrs, 12–24 hrs, 24–36 hrs and 36–44 hrs)

After confirming that azithromycin and analogues have both invasion (Table 1, Additional file 2: Figure S1) and intracellular (Fig. 3a) blood-stage quick-killing activity that is independent of apicoplast-targeting delayed death (Additional file 4: Figure S3a-c), we next determined drug activity across early rings (0–12 hrs post invasion), early trophozoites (12–24 hrs post invasion), late trophozoites (24–36 hrs post invasion) and schizonts (36–44 hrs post invasion). Azithromycin demonstrated a similar IC_50_ across each pulsed treatment stage (0–12 hr IC_50_ 14 μM, 12–24 hr IC_50_ 16 μM, 24–36 hr IC_50_ 15 μM) with these values similar to the IC_50_ values obtained for 44 hr (IC_50_ 11.3 μM) and invasion inhibition (IC_50_ 10 μM) treatments (Fig. 3b, c). We confirmed that azithromycin’s quick-killing activity works rapidly by assessing the morphological effects of pulsed treatment with a 2× IC_90_ drug concentration. Ring-stage treatments (0–12 hrs) showed pronounced vacuolation of the cytoplasm, a typical sign of parasite stress. Trophozoite stages (12–24 hrs and 24–36 hrs) appeared either pyknotic or severely vacuolated with only a 12-hr treatment, indicative of rapid cell death (Fig. 3b, e). Although azithromycin treated schizont stages (36–44 hrs post-invasion) did not show potent growth inhibitory activity when assessed by flow cytometry, light microscopy smears showed late-stage parasites with severe vacuolation and minimal merozoite maturation, indicating this population was indeed killed by azithromycin treatment (Fig. 3e). These data, together with our earlier data, provide direct evidence that azithromycin acts broadly across invasion and throughout the entire blood-stage lifecycle, including early ring stages.

### Azithromycin and analogues rapidly kill early ring-stage parasites

Our finding that azithromycin could kill ring-stage parasites (0–12 hrs post invasion) with similar efficacy to 44 hrs of drug treatment is of major interest since the majority of clinically used antimalarials, with the notable exception of the artemisinins [37, 38], have relatively poor activity against newly invaded ring stages [39–42].

**Fig. 4.**
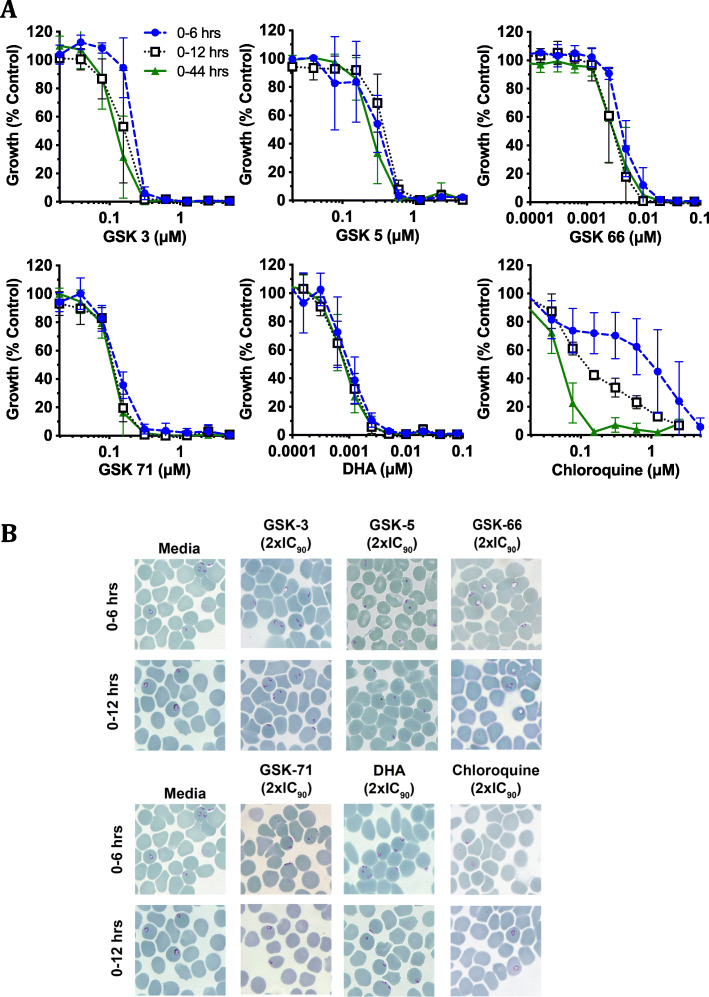
Growth inhibition profiles of azithromycin analogues and control drugs with short-term and in-cycle drug treatments. **a** Early ring-stage *P. falciparum* parasites (0–4 hrs post-invasion) were treated with doubling dilutions of azithromycin analogues/control drugs for 0–6 hrs and 0–12 hrs prior to washing the drug out of cultures allowing growth to continue until parasites were 44 hrs old. A 0–44 hr continuous drug control treatment was also included. **a** Growth inhibition profile of GSK-3 (naphthalene), GSK-5 (substituted phenyl), GSK-66 (chloroquinoline), GSK-71 (quinoline), dihydroartemisinin (DHA) and chloroquine with very early ring-stage treatment across 0–6 hrs and 0–12 hrs post-invasion compared to a full in-cycle treatment. There was no significant difference in drug efficacy between the treatment times of GSK-5 or GSK-71 (*P* > 0.01). GSK-66 showed a significant difference between 0-6 hr vs 0–12 hr treatments (*P* < 0.0079) and 0–6 hr vs 0–44 hr (*P* = 0.001), but there was no significant difference in drug efficacy between 0-12 hr vs 0–44 hr treatments (*P* = 0.96). GSK-3 and DHA showed no significant difference in efficacy between treatment times (*P* > 0.01), with the exception of 0–6 hr vs 0–44 hr (*P* = 0.005 and *P* = 0.01, respectively). In contrast, chloroquine demonstrated a significant difference in drug efficacy between all treatment times (0–6 hr vs 0–12 hr *P* < 0.0001; 0–6 hr vs 0–44 hr *P* < 0.0001; 0–12 hr vs 0–44 hr *P* < 0.0001). Parasitemia was measured via flow cytometry 44 hrs post-invasion. Data represents the means of 3 or more experiments expressed as a percentage of non-inhibitory control and error bars represent ± SEM. Dose response IC_50_s compared using extra sum of squares *F*-test. Repeat measure data is available in Additional file 15 Supporting Value Data. **b** Representative Giemsa-stained thin blood smears showing the growth phenotypes seen for non-inhibitory media controls, and treatment with 2× IC_90_ of azithromycin analogues GSK-3 (0.74 μM), GSK-5 (0.62 μM), GSK-66 (0.034 μM), GSK-71 (0.18 μM) and control drugs DHA (0.003 μM) and chloroquine (0.222 μM) (bottom panels) 0–6 hrs post treatment and 0–12 hrs post treatment

To provide further insights into how quickly azithromycin and analogues act against early ring stages, we examined activity of 6- and 12-hr treatments of early ring stages (0–6 hrs and 0–12 hrs post-invasion treatments) for azithromycin and a panel of diverse analogues that had activity at nanomolar concentrations in parallel. Azithromycin and the analogues tested showed < 2-fold reduction in potency with a 6-hr early ring-stage treatment compared to a 12-hr ring-stage or full 1 cycle (44 hr) treatment, highlighting the drug efficacy against early ring stages (Fig. 3d, e, Fig. 4a, b, Additional file 5: Table S2). Consistent with previous publications, dihydroartemisinin (DHA) treatment resulted in severe growth retardation with early ring-stage treatment [37–39]. DHA is considered to be one of the few clinically used antimalarials with reasonable efficacy against early ring-stage parasites [37–39], making the ability of azithromycin and analogues to also cause rapid death of these stages a promising finding. In contrast, chloroquine had comparatively poor activity for early ring-stage treatments, which is as expected since chloroquine is known to lack potency against ring-stage parasites.

Microscopy analysis was performed for parasites treated with a 2× IC_90_ (0–44 hrs) of azithromycin and analogues to examine the phenotypic changes associated with early ring-stage drug treatment (Fig. 4b). Early (0–6 hrs) ring stages treated with azithromycin GSK-66 and GSK-3 exhibited vacuolation, with evidence of pyknotic cells developing with extended treatment for GSK-71 and GSK-3 (0–12 hrs). Notably, GSK-5 resulted in a large number of pyknotic parasites within only 6 hrs of drug treatment, highlighting the speed with which these compounds can act. DHA treatment of early (0–6 hrs) ring stages did not lead to a clear change in parasite morphology. However, after extended ring-stage treatment (0–12 hrs) pyknotic cells became prominent. No aberrant growth phenotype was observed with chloroquine with treatment of early ring stages (0–6 hrs), with evidence of vacuolation only occurring after extended ring-stage treatment (0–12 hrs). Short-term pulse treatments confirmed that azithromycin and analogues rapidly kill early ring-stage parasites, the growth inhibitory effects are not reversible, and modification of azithromycin can produce analogues with broad and potent efficacy across blood-stage parasite growth.

### Quick-killing azithromycin analogues maintain activity against drug-resistant *P. falciparum* and *P. knowlesi*

We next investigated whether analogues retained potency against the chloroquine/mefloquine/pyrimethamine-resistant *P. falciparum* DD2 line [43], and an artemisinin-resistant *P. falciparum* Cambodian isolate [44–46] (Table 1). Relative to the chloroquine sensitive D10-PfPHG line, DD2 parasites exhibited a 0.24- to 8.4-fold loss of sensitivity to azithromycin and analogues. Of note, analogues featuring a chloroquinoline moiety (GSK-1, GSK-56, GSK-66, GSK-72) were 4.77-fold less potent against chloroquine-resistant DD2, whereas analogues featuring quinoline-, naphthalene- and phenyl-substituted moieties were on average 1.35-fold less sensitive (*n* = 11 compounds) (Table 1, Additional file 6: Table S3).

**Fig. 5.**
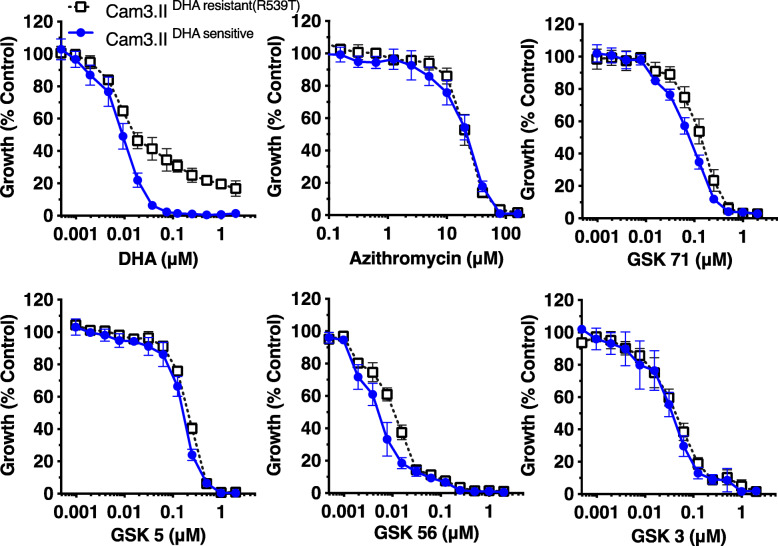
Activity of azithromycin analogues against artemisinin-resistant parasites. Lead azithromycin analogues were tested against artemisinin-resistant Cam3.II^DHA resistant(R539T)^ parasites containing the K13 propeller mutation and reverted, artemisinin-sensitive, Cam3.II^sensitive^ parasites in ring-stage survival assays (4 hr drug pulse of very early rings 0–3 hrs post invasion) prior to washing off drug and assessment of parasitaemia (66 hrs later by flow cytometry). Dihydroartemisinin (DHA), azithromycin, GSK-3 (naphthalene), GSK-5 (substituted phenyl), GSK-56 (chloroquinoline) and GSK-71 (quinoline). Parasitemia was measured via flow cytometry ~ 72 hrs post-invasion. Data represents the mean of 2 or more experiments expressed as a percentage of non-inhibitory control and error bars represent ± SEM. Repeat measure data is available in Additional file 15 Supporting Value Data

We next tested the efficacy of azithromycin analogues against the *P. falciparum* artemisinin-resistant clinical isolate Cam3.II, which has a mutation within the *Kelch13* (PF3D7_1343700) propeller gene (R539T, Cam3.II^DHA resistant(R539T)^) associated with increased early ring-stage (0–3 hrs) survival in vitro with DHA treatment [44–46]. Early ring-stage Cam3.II^DHA resistant(R539T)^ resistant and a reverted sensitive line (Cam3.II^sensitive^) were pulsed for 4 hrs before the drug was washed off, with growth determined 66 hrs later via flow cytometry [46, 47]. Since comparison of IC_50_ has limited relevance in ring-stage survival assays, we compared instead the percentage (%) parasite growth of Cam3.II^DHA resistant(R539T)^ parasites at the drug concentration that inhibited 95% of growth for the Cam3.II^sensitive^ line. As expected, ~ 41% Cam3.II^DHA resistant(R539T)^ parasites survived DHA treatment at the concentration that killed 95% of Cam3.II^sensitive^ parasites (Fig. 5, Table 2). In contrast, growth of both the Cam3.II^DHA resistant(R539T)^ and the Cam3.II^sensitive^ lines were equally inhibited at the concentration that killed 95% of DHA-sensitive parasites for azithromycin and analogues GSK-56, GSK-71, GSK-3 and GSK-5.

**Table 2:** Ring-stage survival assay percent survival values from drug treated artemisinin-resistant and artemisinin-sensitive parasites

Modification	Compound	Cam3.II^sensitive^ 72 hr growth IC_50_ (μM, ±SEM)	Cam3.II^resistant^ 72 hr growth IC_50_ (μM, ±SEM)	Concentration of drug = 5% growth of Cam3.II^sensitive^ (μM)	Growth Cam3.II^DHA resistant(R539T)^ (%)
	DHA	0.007 *(0.002)*	0.011 *(0.001)*	0.05	41
	Azithromycin	30 *(5.5)*	30 *(0.005)*	100	1
Naphthalene	3	0.035 *(0.004)*	0.04 *(0.004)*	0.4	8
2-Chlorophenyl	5	0.2 *(0.02)*	0.28 *(0.005)*	0.5	6
7-Chloroquinoline	56	0.004 *(0.001)*	0.009 *(0.001)*	0.055	7
Quinolone	71	0.07 *(0.007)*	0.15 *(0.03)*	0.6	6

The μM concentration of drug (DHA, azithromycin, GSK-3, GSK-5, GSK-56 and GSK-71) that resulted in a 5% survival value for artemisinin-sensitive Cam3.II^sensitive^ parasites was then used to treat artemisinin-resistant Cam3.II^DHA resistant(R539T)^ parasites, and the resulting % parasite survival value for the resistant parasites is displayed in the table. The IC_50_ value of the drugs against Cam3.II^sensitive^ and Cam3.II^resistant^ strains is also shown to indicate their overall potency against artemisinin-sensitive and artemisinin-resistant parasites. Parasites were incubated for one cycle (72 hrs) after pulsed drug treatment and washing prior to measurement of parasitaemia by flow cytometry. Data represent the mean of 2 experiments

Of note, the IC_50_ of 4 hr ring-stage treatments observed for the Cam3.II^sensitive^ line was similar to that of 6 hr ring-stage treatment seen in D10-PfPHG line upon treatment of azithromycin (Cam3.II^sensitive^ IC_50_ 31 μM, D10-PfPHG IC_50_ 30 μM) and GSK-5 (Cam3.II^sensitive^ IC_50_ 0.20 μM, D10-PfPHG IC_50_ 0.3 μM). Furthermore, activity against early ring-stage Cam3.II^sensitive^ parasites was also similar to the in-cycle (44 hr) treatment activity against D10-PfPHG parasites for azithromycin (Cam3.II^sensitive^ IC_50_ 31 μM, D10-PfPHG IC_50_ 14 μM), GSK-5 (Cam3.II^sensitive^ IC_50_ 0.20 μM, D10-PfPHG IC_50_ 0.26 μM), GSK-56 (Cam3.II^sensitive^ IC_50_ 0.006 μM, D10-PfPHG IC_50_ 0.010 μM), GSK-58 (Cam3.II^sensitive^ IC_50_ 0.075 μM, D10-PfPHG IC_50_ 0.048 μM) and GSK-4 (Cam3.II^sensitive^ IC_50_ 0.04 μM, D10-PfPHG IC_50_ 0.19 μM). Despite the much more stringent drug washout procedure employed for the Cam3.II^sensitive^ ring-stage survival assays, activity against early ring stages was equivalent to that seen for 6 hr treatment of D10-PfPHG ring stages and similar to in-cycle treatments of D10-PfPHG. These results support that azithromycin and analogues have rapid activity against early ring-stage parasites of different *P. falciparum* lines.

We next tested the activity of azithromycin and analogues against the zoonotic malaria parasite *P. knowlesi*, which is a significant human pathogen in regions of Southeast Asia [48] and an in vitro culturable model for *P. vivax* [49]. We found that azithromycin maintains potency against *P. knowlesi* in both in-cycle (28 hr for *P. knowlesi*, *Pk*) and delayed-death (92 hr) assays compared to *P. falciparum* (*Pf*) (*Pk* in-cycle IC_50_ 13 μM, delayed-death IC_50_ 0.08 μM, *Pf* in-cycle IC_50_ 11.3 μM, delayed-death IC_50_ 0.07 μM) (Table 1) as previously shown [50]. We next tested a panel of azithromycin analogues that had potent quick-killing activity against *P. falciparum* for their efficacy against *P. knowlesi* and identified that the majority of analogues had similar quick-killing potency against this divergent parasite species (Additional file 7: Table S4). Of interest, the analogue GSK-9 exhibited a significant 33.1-fold improvement in activity against *P. knowlesi* when compared to activity against *P. falciparum*, suggesting that some species-specific differences in drug activity can occur. Together, these data support that azithromycin analogues have efficacy against diverse human malaria parasites and across DHA and multi-drug-resistant parasites.

### Analogues modified at the macrolactone-ring maintain dual mechanisms of action

We next sought to define whether the more potent quick-killing azithromycin analogues maintained apicoplast-targeting delayed-death activity. As quick-killing IC_50_s for a number of analogues (GSK-1, GSK-4, GSK-5, GSK-29, GSK-57, GSK-66, GSK-71, GSK-78) approached that of the delayed-death IC_50_ values of azithromycin (120 hr IC_50_ 0.07 μM), the measurement of apicoplast targeting delayed-death activity (i.e. activity after 120 hrs of treatment, Fig. 1d) would likely be compromised by quick-killing potency. Therefore, we assessed the activity of azithromycin and a panel of quick-killing analogues against the azithromycin-sensitive bacteria *Streptococcus pneumoniae* (Additional file 8: Table S5) on the basis that this Gram-positive bacteria’s ribosome could serve as a proxy for the malaria parasite bacterium-like apicoplast ribosome [12, 51]. Consistent with previously published results, limited inhibition of bacterial growth was observed for analogues with an N-substitution on the desosamine sugar moiety [34, 35, 52]. Indeed, N-substituted analogues of azithromycin have been deliberately designed to reduce off-target drug activity against bacteria for use in alternative drug applications [34, 35, 52]. In contrast, all analogues with N6-substitutions on the macrolactone backbone (GSK-1, GSK-4, GSK-5, GSK-6, GSK-9, GSK-11, GSK-12, GSK-16, GSK-17, GSK-21, GSK-25) had activity against *S. pneumoniae* similar to azithromycin. Thus, selecting the site of azithromycin modification can allow improved quick-killing activity while maintaining apicoplast targeting delayed-death activity, or delayed-death activity can be removed along with off-target antibacterial effects to produce a quick-killing specific antimalarial.

### Analysis of the quick-killing mechanism of action suggests a multi-factorial mechanism of action

In an attempt to identify the molecular target of quick-killing activity, we selected for in vitro drug resistance by subjecting an azithromycin delayed-death-resistant D10 line (D10-AZR^r^) with a stepwise increase [12] of the quick-killing azithromycin analogue GSK-59 featuring a chloroquinoline-substituted desosamine moiety that lacks delayed-death activity. After three attempts, we failed to select for resistant parasites > 3 months after drug removal, suggesting that the mechanism of quick-killing cannot be readily selected for in vitro.

**Fig. 6.**
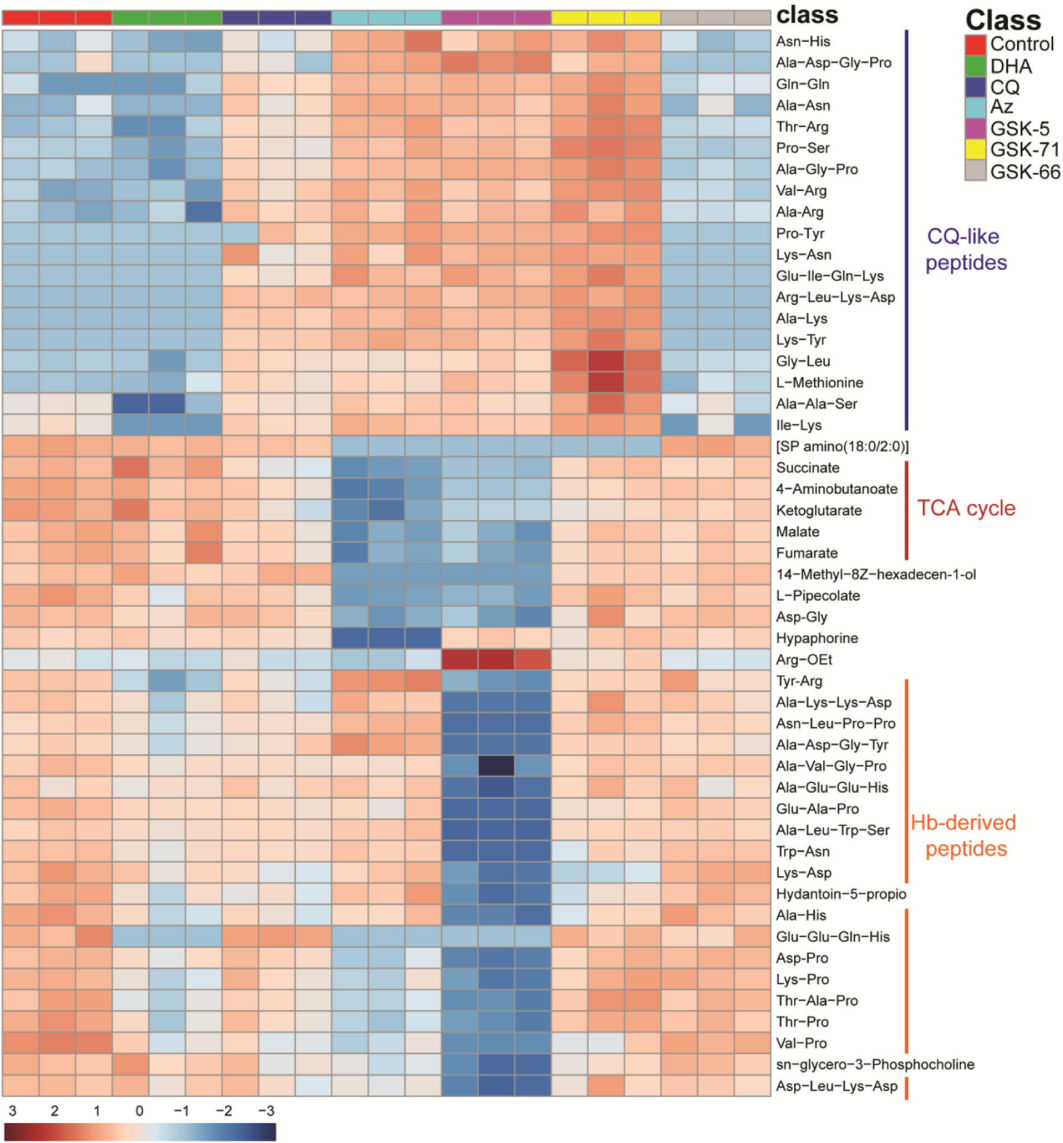
Hierarchical clustering of the different sample groups, treated with chloroquine (CQ) (blue), DHA (green), azithromycin (Az) (light blue), GSK-5 (purple), GSK-71 (yellow), GSK-66 (grey) and ethanol control (red). Vertical clustering displays similarities between sample groups, while horizontal clusters reveal the relative abundances of the 50 most significantly different metabolites from experiment 1. The significantly differentially regulated metabolites are further classified into three different groups, the CQ-like peptides (blue line), TCA cycle (red line) and haemoglobin-derived peptides (orange lines). All compounds were tested with three technical replicates. White indicates no change, while red and blue indicates increased and decreased abundances respectively. Ward’s minimum variance method algorithm was used to generate the hierarchical cluster analysis

**Fig. 7.**
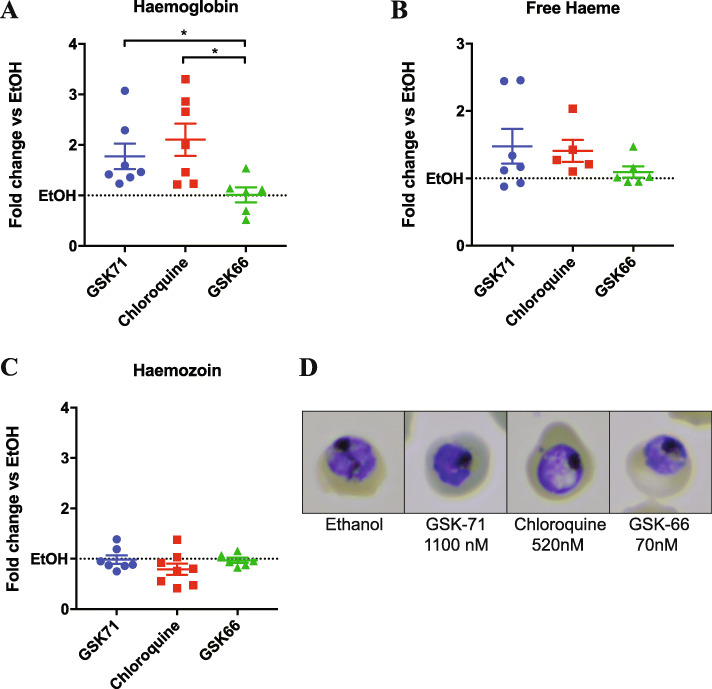
Haemoglobin fractionation of GSK-71, chloroquine and GSK-66-treated *Plasmodium falciparum* (3D7) parasites compared to an ethanol control. Scatter dot plots representing the relative levels of **a** haemoglobin, **b** free haem and **c** haemozoin in trophozoite-stage parasites following a 5 hr incubation with 10× IC_50_ (44 hr) concentration of GSK-71 (1100 nM), chloroquine (520 nM) and GSK-66 (70 nM) expressed as the fold change when compared to an EtOH control. Data is represented as the mean of > 3 paired replicates from three independent experiments with the error bars expressed as SEM. Significant differences were assessed using Student’s *t* test. Repeat measure data is available in Additional file 15 Supporting Value Data. **d** A panel of representative Giemsa-stained parasites treated with 10× IC_50_ (44 hr) concentration of GSK-71, chloroquine, GSK-66 and the ethanol negative control after 5 hrs.

We next undertook an untargeted metabolomics screen to identify changes in the metabolomic signature of azithromycin and the quick-killing analogues GSK-5 (substituted phenyl), GSK-66 (chloroquinoline) and GSK-71 (quinoline) and to compare changes during treatment with these analogues to known antimalarials, such as chloroquine and DHA (Fig. 6, Additional file 9: Figure S4, Additional file 10: Table S6, Additional file 11: Table S7, Additional file 12: Table S8). Following a 2 hr treatment of trophozoite-stage parasites at a 5× IC_50_ (44 hr) concentration, supervised multivariate analysis (partial least squares-discriminate analysis) and heat map showed that the most prominent metabolomic signature shared between azithromycin and analogues was a series of short peptides that were increased for all of azithromycin, GSK-71, GSK-5 and the food vacuole-targeting control drug chloroquine (Fig. 6, Additional file 10: Table S6a&b). Since increases in these peptides have previously been demonstrated for chloroquine- and piperaquine-treated trophozoites [53], it is possible that this signature indicates a mechanism of action similar to the 4-aminoquinolines, which are thought to act by inhibiting crystallisation of haemoglobin-derived haem to form haemozoin within the parasite’s food vacuole. However, it was also noted in the study by Creek et al. that the sequences for the majority of these peptides are not derived from degraded haemoglobin, indicating that the metabolomic signature shared between chloroquine, azithromycin, GSK-71 and GSK-5 are likely due to disruption of proteolytic processes other than haemoglobin digestion. In addition, GSK-66 which has the most chloroquine-like functional group in terms of structure and was the most potent analogue tested in this study, showed little in the way of changed metabolites and gave a profile most similar to untreated control. Since chloroquine is known to disrupt the haemoglobin digestion pathway by inhibition of haemozoin formation [54–56], we next measured the levels of haemoglobin, haem and haemozoin in the parasites following treatment with analogues GSK-66 (chloroquinoline) and GSK-71 (quinoline) [57] (Fig. 7). Trophozoite-stage parasites were treated with CQ, GSK-71, and GSK-66 at 10× IC_50_ for 5 hrs. There was an increase in measurable haemoglobin and a reduction in haemozoin formation for parasites treated with chloroquine, as expected for this known inhibitor of haemoglobin digestion and haemozoin formation. A similar build-up in haemoglobin was seen for GSK-71; however, there was no decrease in haemozoin, supporting that this drug may have activity in the food vacuole, but this did not involve measurable inhibition of haemozoin formation. Again, GSK-66 treatment had no effect on haemoglobin or haemozoin levels, supporting the non-targeted metabolomics data which suggests that this drug has limited effects on parasite metabolism at the concentration and duration tested. These data support that azithromycin and analogues have activity in the food vacuole of drug-treated trophozoites, but also indicate additional activity outside of haemoglobin digestion.

A second shared metabolomic signature was observed for azithromycin and the phenyl-substituted analogue GSK-5, with a major reduction in key metabolites (including succinate, fumarate, malate) of the mitochondrial tricarboxylic acid (TCA) cycle (Additional file 11: Table S7a&b, Additional file 13: Figure S5). The reduction in TCA metabolites was evident across repeat experiments for azithromycin, but was less prominent for GSK-5 in the second experiment (Additional file 11: Table S7a&b). Although several steps in the *Plasmodium* TCA cycle are considered dispensable in blood-stage parasites, the fumarate hydratase conversion of fumarate to malate followed by the malate quinone oxidoreductase (MQO) mediated conversion of malate to oxaloacetate are thought to have important roles in the parasite’s purine salvage pathway [58, 59]. Reduced bioavailability of fumarate and malate, two key metabolites required for efficient purine salvage, would negatively impact on purine production and parasite growth over time and offers a novel drug development strategy. Indeed, a recent paper has identified blood-stage inhibitors of MQO in the Pathogen Box [60] suggesting that this pathway is a viable drug target against asexual-stage parasites. These data implicate a second membrane-bound organelle as a potential target during trophozoite stages of the parasite lifecycle, underlining the potential for multifactorial mechanisms of action.

Azithromycin and GSK-5 also caused a reduction in haemoglobin-derived peptides across both experiments to levels lower than seen for chloroquine and DHA, two food vacuole targeting drugs (Additional file 12: Table S8a&b). Thus, treatment with azithromycin and GSK-5 caused an increase in specific non-haemoglobin-derived peptides similar to that seen for chloroquine, a consistent decrease in haemoglobin-derived peptides (most prominently for GSK-5 in this data set) and a decrease in TCA cycle metabolites. In contrast, GSK-71 was most notably associated with an increase in non-haemoglobin chloroquine-like peptides, while GSK-66 and DHA had minimal impact on the metabolic profile under the conditions analysed here. This highlights the potential ability of azithromycin analogues with different structures to interrupt normal metabolic functions across the cell and in different organelles, even when used at the same fold-IC_50_ and against the same lifecycle stages.

Given the metabolomics evidence suggesting that azithromycin and analogues may target the food vacuole, we investigated whether the rapid ring-stage killing activity of the chloroquinoline analogue GSK-66 (Fig. 4a, b, Table 1) may be a result of azithromycin pre-sensitising ring stages to the chloroquinoline moiety. We treated early ring-stage D10-PfPHG parasites (0–6 hrs) with azithromycin at an IC_10_ concentration and added a dilution series of chloroquine. Addition of azithromycin did not potentiate chloroquine’s activity against early ring stages, with the IC_50_ of azithromycin+chloroquine remaining well above the activity of GSK-66 (Additional file 14: Figure S6). In addition, a range of functional groups were found to potentiate azithromycin’s quick-killing activity. These combined data suggest that azithromycin does not pre-sensitise parasites to chloroquinoline-like moieties nor act through disruption of haem polymerisation per se as chloroquine is believed to, but rather may act more broadly within the parasite’s food vacuole as well as potentially other cellular and organellar targets such as the parasite’s mitochondrion.

## Discussion

The spread of parasites resistant to artemisinin combination therapies (ACTs) in Southeast Asia, India and other regions highlights the need for novel antimalarial drug treatment strategies to ensure timely and effective treatment of clinical disease [3–6, 8]. Despite limited use against clinical cases of malaria, macrolide antibiotics remain of interest as potential partner drugs in antimalarial combinations due to their activity against malaria parasites and well-established safety profile in children and pregnant women [10, 11, 24, 61]. Recently, we identified that high concentrations of clinically used macrolides inhibit merozoite invasion in vitro and showed that this mechanism of action was independent of apicoplast-targeting delayed death [29]. Here, we demonstrate the potential for the antibiotic azithromycin to be repurposed as an antimalarial with two potent mechanisms of action with the identification of azithromycin analogues that have potent activity throughout intra-erythrocytic parasite development and against merozoite invasion. We established that this activity is through a mechanism independent of the known activity of azithromycin against the parasite apicoplast, revealing potential new pathways for development of novel antimalarials.

We investigated the activity of a panel of the analogues and identified 65 with improved in-cycle activity (44 hr early rings to schizont treatment) compared to azithromycin. Of these, 39 analogues with diverse functional groups including substituted phenyl (GSK-5, IC_50_ 0.02 μM), naphthalene (GSK-3, IC_50_ 0.183 μM), quinoline (GSK-58, IC_50_ 0.048 μM) and chloroquinoline (GSK-66, IC_50_ 0.007 μM) had nanomolar IC_50_s, providing between an 11- to 1615-fold improvement over azithromycin.

Azithromycin and analogues exhibited equipotent quick-killing activity across intracellular blood-stage parasite growth. This included rapid activity against early ring-stage development (both 0–6 and 0–12 hrs post invasion) at a similar potency to 0–44 hr (one cycle) treatments. Therefore, azithromycin and analogues have a similar efficacy profile to the artemisinins [37, 38], being effective against early ring stages and across the blood-stage lifecycle, but with additional potential to be active against liver and transmission-stage parasites [22, 26, 27]. We found that the azithromycin analogues with the best activity in 44 hr assays (GSK-3, GSK-5, GSK-56 and GSK-72) also exhibited the greatest improvement in invasion inhibitory activity over azithromycin, highlighting that both quick-killing activities can be improved over azithromycin. However, the ability to push potency of merozoite invasion inhibition into clinically relevant concentrations below 1 μM may be limited. Importantly, assays where merozoites were treated directly prior to compound removal and addition of RBCs to begin invasion show that the invasion inhibitory activity of azithromycin and analogues is directed against the merozoite and not against the RBC. A number of invasion inhibitory antimalarial strategies are being pursued globally (reviewed in [62]), and there remains the possibility that further improvements in azithromycin analogue invasion inhibitory IC_50_ are achievable with additional development.

It is interesting to note that improved quick-killing activity is ubiquitous across analogues with phenyl, naphthalene, quinoline and chloroquinoline functional groups. It has previously been hypothesised that the high potency of several analogues featuring quinoline and chloroquinoline moieties was due to these analogues acting like hybrid azithromycin (apicoplast ribosome targeting) and chloroquine (food vacuole target) activity [33, 34] molecules. Interestingly, azithromycin analogues with the four functional groups display properties dissimilar to chloroquine, these being (i) improved invasion inhibitory activity compared to azithromycin, whereas chloroquine does not inhibit invasion [39, 63], and (ii) similar activity against chloroquine-resistant and chloroquine-sensitive lines for analogues featuring substituted phenyl, naphthalene and quinoline moieties. Activity against chloroquine-resistant DD2 for analogues with chloroquinoline functional groups was variable with two analogues showing improved potency against the chloroquine-resistant line over the chloroquine-sensitive line, while three compounds were less potent against the resistant line; and (iii) potent inhibition of very early ring stages (0–6 hrs post invasion), which are largely insensitive to chloroquine. However, additional evidence from this study does support the idea that azithromycin and analogues quick-killing activity may, in part, be acting against the parasite’s food vacuole.

Although our ability to perform comprehensive and detailed SAR comparison was limited by compound availability impacting on matched-pair analysis, some general trends were observed with the analogues available. Analogues with chloroquinoline and quinoline substituents were generally the most potent in one-cycle 44 hr assays. Naphthalene had modest potency and is a close bioisostere of quinoline. In general, analogues with a short carbon linking the amino quinoline to the N6-position of the macrocycle or the *O*- or *N*-position of the desosamine group were the most active. Appending functional moieties to the N6-position of the macrolactone, or to the desosamine sugar, both conferred significantly improved in-cycle activity, with a slight tendency for improved quick-killing activity when the functional group was either attached to the *N*- or the *O*- of the desosamine sugar as opposed to the N6-position of the macrolactone (i.e. chloroquinoline GSK-66^desos^ (IC_50_ 0.007 μM) and GSK-1^macro^ (IC_50_ 0.019 μM); naphthalene GSK-78^desos^ (IC_50_ 0.51 μM) and GSK-12^macro^ (IC_50_ 0.59 μM)). Thus, the position of the functional group on the macrocyclic did not greatly impact activity, suggesting the macrocycle may be acting as a vehicle for transportation of the active functionality.

Within the parasite, it is possible that analogues are metabolised and then release the pendant quinoline or aromatic system as the active component of compound. This is possible either by an oxidative mechanism hydrolysing amine-linked substituents, or by proteolytic or hydrolytic degradation of the amide and urea functionality linking the pendant quinoline or aromatic group to the macrolactone. In this study, we could not conclusively address whether metabolism was occurring, but this will be an important facet to address in a future mechanistic study of these azalide analogues. The possibility of the macrolactone acting as a delivery vehicle with subsequent metabolic release of the active payload in the parasite raises the prospect for the azithromycin scaffold to be tethered to and act as a delivery vehicle for other antimalarials that act at a similar asexual killing rate to chloroquine, akin to antimalarial candidates undergoing clinical trials such as KAF156 or MMV048 [64]. Such a strategy to improve dual target efficacy of azithromycin analogues, and delay the onset of resistance, is an attractive option. Furthermore, while it has been demonstrated that these analogues have efficacy in in vivo rodent models [31, 33, 35], the effective contribution of quick-killing has not been assessed. In addition, whether these analogues would be stable to first pass metabolism in the liver is another important aspect to consider in future development of the azalide analogue class.

Although the azithromycin analogues identified as having improved quick-killing activity in this study feature a range of added functional groups, compounds with quinoline and chloroquinoline moieties feature prominently amongst the most potent quick-killing analogues. Hybrid molecules featuring quinolines fused to a second chemotype with antimalarial properties such as endoperoxides [65] or reversed chloroquine drugs that are linked to a reversal agent, a molecule known to inhibit or circumvent the activity of the chloroquine resistance transporter *Pf*CRT [66, 67], have been developed and shown to have efficacy in rodent malaria models (reviewed in [68]). The current lead reversed chloroquine compound, DM1157 [69], has shown low nanomolar potency against chloroquine-resistant parasites, demonstrated efficacy against *P. chabaudi* rodent malaria parasites and has recently undergone Phase I trials in humans (NCT03490162, [70]). Despite the potential of DM1157, hybrid molecules have faced hurdles in development including examples of endoperoxide hybrids unable to overcome existing resistance mechanisms [71] and the high MW of the compounds impacting on desirable drug-like properties. In this regard, it is interesting to note that the ketolide antibiotics solithromycin and telithromycin, semi-synthetic derivatives of erythromycin which both feature a large functional group added to the macrolactone ring, have been progressed for clinical use. This highlights that modified macrolides can be developed that maintain favourable drug-like properties despite their high MW.

Metabolomic analysis of azithromycin and analogue-treated parasites suggests one potential site of drug activity in trophozoite stages is the parasite’s food vacuole, with a similar build-up of largely non-haemoglobin peptides observed for azithromycin, analogues GSK-5 and GSK-71 as seen for chloroquine. However, a number of differences to chloroquine were also observed including the chloroquinoline-modified analogue GSK-66 causing minimal change in parasite metabolism, azithromycin and GSK-5 having activity against mitochondrial metabolism and GSK-5 also causing a reduction in haemoglobin-derived peptides. Previous studies have shown that trophozoite-stage treatment with the mitochondrial targeting drug atovaquone, alone and in combination with proguanil, leads to a build-up of the TCA metabolite fumarate [53, 72]. It was postulated that this could be a result of the TCA enzyme malate-quinone oxidoreductase complex also having a role in the mitochondrial electron transport chain (the target of atovaquone) that may be affected by atovaquone, leading to off-target disruption of the TCA cycle. In contrast, azithromycin and GSK-5 treatment caused a reduction in fumarate and other TCA metabolites, a signature different to that of atovaquone. Interestingly, treatment with the membrane-bound glucose transporter inhibitor 3361 led to a reduction in TCA and haemoglobin-derived peptides after 6 hrs of drug treatment [72], similar to that seen for azithromycin and GSK-5 here. The multiple changes in parasite metabolic networks seen when inhibiting glucose uptake supports data generated in this study that suggests azithromycin and analogues quick-killing activity may occur through multifactorial mechanisms.

While there are limitations in this analysis, including only one lifecycle stage and drug concentration (5× the 44 hr IC_50_) tested for each analogue, these data clearly demonstrate that azithromycin and analogues likely have multi-factorial mechanisms of action even against a single lifecycle stage. Given the apparent site of activity for azithromycin and analogues includes the membrane-bound food vacuole and mitochondrion, it is possible that additional membrane-bound organelles in other lifecycle stages (i.e. the rhoptry in merozoites) could also be the target of these drugs. Additional experimental validation for the site of activity across a range of analogues and lifecycle stages will need to be undertaken in order to detail the potential promiscuity of these drugs in stopping parasite growth.

Previous studies have suggested that azithromycin analogues may act through a chloroquine-like mechanism [33–35] (reviewed in [73]), and evidence presented in this study from metabolomic experiments and haemoglobin fractionation assays supports that one of the sites of activity for azithromycin and analogues is the parasite’s food vacuole. If a chloroquine-like targeting of the food vacuole is an important component of azithromycin and analogue quick-killing activity, these modified analogues have two major advantages over chloroquine and quinine for clinical treatment. Firstly, phenyl-, naphthalene- and quinoline-substituted analogues maintained reasonable activity against chloroquine-resistant DD2 parasites. The maintenance of potency against chloroquine-resistant parasites could be explained by the different properties of the drug limiting the ability of the mutated chloroquine-resistant transporter to expel the drug from the developing vacuole [74, 75]. Secondly, azithromycin and analogues have rapid activity against early ring-stage parasites. Rapid activity against ring stages is in stark contrast to the poor activity of chloroquine and quinine against these early parasites and it is certainly possible that azithromycin and analogues could access the site of the initial stages of haemoglobin digestion, similar to artemisinin [37, 38, 76], via superior lipophilic properties [33, 34].

Azithromycin and analogues display several other properties of interest. The majority of quick-killing analogues tested against chloroquine/pyrimethamine-resistant DD2 [43] and artemisinin-resistant Cam3.II^DHA resistant(R539T)^ [44, 45] retained potency compared to the chloroquine- and artemisinin-sensitive D10-PfPHG and artemisinin-sensitive Cam3.II^sensitive^ lines. While there were examples of chloroquinoline containing analogues being less potent against DD2 parasites, these data broadly indicate that a wide range of azithromycin analogue modifications can significantly improve quick-killing activity in a way that overcomes a number of established resistance mechanisms. Azithromycin and analogue invasion blocking activity is shared across distantly related Apicomplexan parasites such as *Toxoplasma gondii* [29, 77], *P. berghei* [29] and the zoonotic human malaria parasite *P. knowlesi*. Since neither *T. gondii* nor *Plasmodium* spp. merozoites contain a food vacuole, the target of chloroquine, it seems likely that azithromycin and analogues have additional mechanisms of action, with properties such as modulation of intraerythrocytic calcium (Ca^2+^), interference of kinase signalling pathways, cationic trapping and sequestration within acidic environments, as well as decreasing mobility of phospholipid bilayers demonstrated for azithromycin in other eukaryotic cell systems, all potential alternative MOAs contributing to quick-killing [78–82].

Finally, the influence of the site of modification to azithromycin and the addition of different functional groups was investigated in the context of delayed-death activity. Previous studies have demonstrated that the desosamine sugar is critical for binding to bacterial ribosomes, and we anticipated that modifications to this region would stop apicoplast-targeting delayed-death activity [12, 51, 52]. However, the potent quick-killing activity of azithromycin analogues (GSK-4, GSK-5, GSK-12, GSK-16, GSK-57, GSK-71, etc.) precluded assessment of delayed-death activity using traditional 120 hr parasite assays. Therefore, we assessed whether a focused set of azithromycin analogues maintained their activity against prokaryotic ribosomes by determining the minimum inhibitory concentration (MIC) activity of the gram-positive bacteria, *S. pneumoniae*. Comparison of *P. falciparum* quick-killing IC_50_ and *S. pneumoniae* MIC confirmed that attaching the functional group to the desosamine sugar (GSK-57, GSK-66, GSK-71 and GSK-78) abrogated activity against bacterial ribosomes as expected. In contrast, analogues with the functional group attached to the N6-positon of the macrolactone (GSK-1, GSK-4, GSK-5, GSK-6, GSK-9, GSK-11, GSK-12, GSK-16, GSK-17, GSK-21, GSK-25) maintained activity against *S. pneumoniae*, suggesting that delayed-death activity via targeting the bacterium-like ribosome of the apicoplast is maintained in analogues featuring modification to the N6-positon of the macrolactone (GSK-1, GSK-4, GSK-5, GSK-6, GSK-9, GSK-11, GSK-12, GSK-16, GSK-17, GSK-21, GSK-25). Thus, analogues could be modified to act through either single (i.e. quick-killing) or dual (i.e. quick-killing and delayed-death) mechanisms of action depending on the properties desired (i.e. quick parasite clearance and/or long-term prophylaxis) and whether removal of non-selective antibiotic activity is preferred over apicoplast-targeting delayed-death prophylaxis.

## Conclusion

We have shown that azithromycin and analogues have a quick-killing mechanism of action that kills parasites throughout intracellular blood-stage development, including inhibition of merozoite invasion of RBCs. Additionally, azithromycin analogues exhibit promising potency against very early ring-stage parasites, which is a rare feature amongst existing antimalarials. Importantly, quick-killing can be improved without losing activity against protein synthesis by the apicoplast ribosome (delayed death). Conversely, the option to engineer azithromycin to remove activity against a bacterium-like ribosome and thereby avoid selection for ‘bystander’ bacterial resistance is available. Further development of azithromycin analogues offers the prospect of designing compounds with either quick-killing (quick-parasite clearance) mode of action or both quick-killing and slow-killing prophylactic activity. This design strategy should also retard resistance acquisition by hitting two targets. Fine-tuning the quick-killing activity of azithromycin analogues significantly broadens its clinical applications and offers resistance proofing through two independent mechanisms of action. Therefore, the identification of potent azithromycin analogues with rapid killing phenotypes and dual mechanisms of action (delayed-death and quick-killing activity) provide a new avenue for anti-malarial drug development.

## Methods

### Antimalarial drugs

Azithromycin analogues (GSK-1–84) were a gift from GlaxoSmithKline and were synthesised as described previously [31–35, 83]. Additional file 1: Tables S1a-c provides further details of chemical structure and analogue origin. Stock concentrations of quinine (3075 mM Sigma), azithromycin (100 mM, AK-Scientific) and GSK analogues (10 mM, GSK-1–84) were made up in ethanol as vehicle. Chloroquine diphosphate salt (10 mM, Sigma-Aldrich) was dissolved in 10% acetic acid in H_2_O. Dihydroartemisinin (10 mM, DHA, Sigma-Aldrich) were dissolved in dimethyl sulfoxide (DMSO). Drugs were added such that the vehicle was diluted > 100-fold for merozoite invasion assays and > 1000-fold for intracellular growth assays to minimise non-specific inhibition.

### Culture and synchronisation of *Plasmodium* spp*.* parasites

Green fluorescent protein (GFP) expressing *P. falciparum* D10-PfPHG parasites [84], DD2 [43], artemisinin-resistant (Cam3.II^DHA resistant(R539T)^) and artemisinin-sensitive (Cam3.II^sensitive^) Cambodian isolates [45] and *P. knowlesi* PkYH1 [85] were cultured in human O^+^ erythrocytes (RBCs) (Australian Red Cross Blood Service). Parasites were cultured in RPMI-HEPES culture medium (pH 7.4, 50 μg/mL hypoxanthine, 25 mM NaHCO_3_, 20 μg/mL gentamicin, 0.5% Albumax II (Thermo Fisher Scientific)) and maintained in an atmosphere of 1% O_2_, 4% CO_2_ and 95% N_2_ according to established protocols [86]. Tight synchronisation of D10-PfPHG parasites was achieved using sodium heparin [63, 87]. *P. falciparum* DD2, the Cambodian isolates and *P. knowlesi* (PkYHI), were synchronised with continuous passage over a gradient of 70% Percoll (Sigma-Aldrich) for purification of late-stage schizonts and 5% w/v sorbitol (Sigma-Aldrich) treatments for ring stages.

### Drug inhibition assays

A diagram outlining the different *Plasmodium* spp*.* drug inhibition assays used in this study is available in Fig. 1 and has been described previously [29, 63]. Stage specificity assessment of azithromycin or analogues during blood-stage *P. falciparum* development was undertaken through the addition of the drug at the specified time points (0–6 hrs, 0–12 hrs, 12–24 hrs, 24–36 hrs or 36–44 hrs post merozoite invasion) and the subsequent removal through three consecutive washes with 200 μl of medium (centrifuged at 300×*g* for 2 min) before resuspending in a final volume of 200 μl. Parasite growth was quantified at late schizont stages (44–48 hrs post invasion) by flow cytometry of parasites stained with 10 μg/mL ethidium bromide (EtBr) for 1 hr prior to washing with PBS.

### Invasion inhibition assays

Purification of viable merozoites and merozoite invasion inhibition assays has been described previously [29, 63, 87]. Briefly, 300 mL of D10-PfPHG schizont culture, 3% haematocrit and 4–5% parasitaemia tightly synchronised to a 6 hr window of invasion with heparin were magnet purified (Mitenyi Biotech) away from RBCs at 40–46 hrs post-invasion. Purified schizonts were eluted in up to 30 mL of media, 10 μM of E64 (Sigma-Aldrich) was added and the parasites were left to mature for 5 hrs. Schizonts were filtered through a 1.2-μm syringe filter (Minisart, Sartorius) in incomplete media with NaHCO_3_ to release merozoites and 22.5 μl of filtrate was added to 2.5 μl of drug prior to addition of RBC (0.5% final haematocrit). Plates were agitated at 400 rpm for 10 min at 37 °C to promote invasion.

For drug washout, 90 μL of purified merozoites was added to 10 μL of either incomplete media (no serum) or incomplete media plus drug before transfer to a 0.22-μm Ultrafree-MC centrifugal filter (Thermo Fisher). Filter columns were centrifuged at 750 rcf for 1 min and washed with incomplete media twice. Free merozoites were resuspended off the filter in 45 μL of incomplete media and transferred to 96-well U-bottom plates containing 5 μL of RBCs at 1% haematocrit (final haematocrit of 0.1%). Plates were agitated at 400 rpm for 10 min at 37 °C and cultures were incubated at 37 °C for 30 min. Cells were treated with 5 μg/mL EtBr for 10 min prior to being washed in 1 x PBS and ring-stage parasitemia measured by flow cytometry.

### Ring-stage survival assays (RSA_0-3h_)

For ring-stage survival assays [44–46], tightly synchronised artemisinin-resistant Cam3.II^DHA resistant(R539T)^ and artemisinin-sensitive Cam3.II^sensitive^ late schizont stage parasites were concentrated over a gradient of 70% Percoll (Sigma-Aldrich), washed once in complete medium and incubated for 3 hrs with fresh RBCs to allow invasion. Cultures were sorbitol treated to eliminate the remaining schizonts. The 0–3 hr post-invasion rings were adjusted to 1% parasitemia and 1% haematocrit before exposure to a serial dilution of DHA, azithromycin and azithromycin analogue concentrations for 4 hrs. Plates were washed five times with 200 μl of medium before parasites were transferred into a new 96-well plate to ensure the complete removal of drug [47]. Parasites were grown for a further 66 hrs, before parasitemia was assessed by flow cytometry.

### Apicoplast-null inhibition assays

Apicoplast-null (D10-PfPHG^apicoplast-null^) [17, 36] parasites were generated through supplementation of culture media with 200 μM isopentenyl pyrophosphate (IPP) and apicoplast removal through treatment with 0.35 μM (5× IC_50_) of azithromycin for 6 days, with parasites cultured continuously thereafter with IPP. Removal of the apicoplast was confirmed by growing D10-PfPHG^wildtype^ and D10-PfPHG^apicoplast-null^ (+IPP) parasites with reducing concentrations of azithromycin for ~ 120 hrs which identified a ~ 64 fold-change in the IC_50_ values between the parasite populations (D10-PfPHG^apicoplast-null^ IC_50_, 4.5 μM; D10-PfPHG^wildtype^ IC_50_, 0.07 μM) confirming apicoplast removal. To test for azithromycin analogue activity against the apicoplast, tightly synchronised ring-stage D10-PfPHG^apicoplast-null^ (+IPP) and D10-PfPHG^wildtype^ parasites were treated with the in-cycle 90% inhibitory concentration (IC_90_) of drugs obtained for D10-PfPHG^wildtype^ for ~ 44 hrs (in-cycle) and the resulting growth inhibition determined by flow cytometry.

### Flow cytometry and microscopy analysis of inhibition

Parasitaemia was measured on an LSR Fortessa (Becton Dickinson) with a 96-well plate reader. Mature (> 36 hr post-invasion) *P. falciparum* D10-PfPHG parasites were counted using Fl-1-high (GFP; excitation wavelength, 488 nm) and Fl-2-high (EtBr; excitation wavelength, 488 nm). D10-PfPHG ring-stage parasites (< 6 hrs post invasion) were counted using a Fl-1-high (GFP) and Fl-2-low (EtBr) gate [63]. Mature parasites of the remaining lines were gated with a forward scatter (FSC) and FL-2-high (EtBr) gate [63]. Typically, 20,000–40,000 RBCs were counted in each well. Samples were analysed using FlowJo software (TreeStar Inc) with growth of drug treatments normalised against media control wells to calculate the percentage survival. Thin smears for microscopy were fixed with fresh methanol and stained in 10% Giemsa (Merck) for 10 min. IC_50_s and IC_90_s were determined for each drug using GraphPad Prism (GraphPad Software) according to the recommended protocol for nonlinear regression (constrained to top = 100 and bottom = 0) of a log-(inhibitor)-versus-response curve.

### Selection of azithromycin-resistant *P. falciparum* lines

In vitro selection of quick-killing-resistant lines was carried out using a *P. falciparum* (D10-PfPHG) line featuring a G91D mutation in the apicoplast ribosomal gene, *rpl4*, resulting in a ~ 57-fold loss of sensitivity to azithromycin’s delayed-death activity (2 cycles, Fig. 1d) (D10-AZR^r^). To select for quick-killing resistance [12], D10-AZR^r^ parasites were first exposed to 3× IC_50_ of GSK-59 (chloroquinoline moiety, delayed-death inactive drug) for 3 days, followed by a 5× IC_50_ concentration for 4 days then 3× IC_50_ for an additional 2 days prior to removal of the drug. After treatment, parasites were fed once every 2 days, and once a week, 30–40% of culture was replaced with fresh RBCs. Parasites were examined every 2 to 3 days by Giemsa-stained thin blood films for between 3 (90 days) and 5 months (150 days) with no recrudescent parasites observed.

### Antibacterial screen

Antibacterial activity of azithromycin and analogues against *Streptococcus pneumoniae* was determined using 96-well minimum inhibitory concentration (MIC) assays [88]. Two-fold serial dilutions were added to macrolide-sensitive D39 *S. pneumoniae* in 100 μL Mueller Hinton Broth supplemented with 5% lysed horse blood. Bacterial growth was assessed after 24 hrs incubation with drug by estimating the MIC where bacterial growth, as indicated by a media colour change, could be identified (MIC expressed as μM).

### Sample extraction for metabolomics analysis

For metabolomics experiments, two 150-mL flasks at 6% haematocrit containing tightly synchronised ~ 30–34 hr D10-PfPHG trophozoites were harvested via magnet purification (Miltenyi Biotech). Infected RBC density was quantitated by flow cytometry [89], and 2 mL of 3 × 10^7^ parasites were added to and incubated in 24-well microtiter plates for 1 hr at 37 °C to stabilise the culture. Drugs (5× IC_50_) were added and incubated for a further 2 hrs prior to removal of the supernatant, 2× washes with 800 μL ice-cold 1× PBS with cells pelleted via centrifugation at 400×*g* for 5 min at 4 °C. The cell pellets were resuspended in 150 μL of ice-cold extraction buffer (MeOH) containing 1 μM internal standards; CHAPS and PIPES, and incubated on ice for 1 hr with shaking at 200 rpm. Insoluble material was pelleted with centrifugation at 14,800×*g* for 10 min at 4 °C and 120 μL of supernatant was collected and stored at − 80 °C until analysis.

### LC-MS analysis

Liquid chromatography-mass spectrometry (LC-MS) data was acquired on a Q-Exactive Orbitrap mass spectrometer (Thermo Scientific) coupled with high-performance liquid chromatography system (HPLC, Dionex Ultimate® 3000 RS, Thermo Scientific) as previously described [53]. Briefly, chromatographic separation was performed on a ZIC-pHILIC column equipped with a guard (5 μm, 4.6 × 150 mm, SeQuant®, Merck). The mobile phase (A) was 20 mM ammonium carbonate (Sigma Aldrich), and (B) acetonitrile (Burdick and Jackson) and needle wash solution was 50% isopropanol. The column flow rate was maintained at 0.3 ml/min with temperature at 25 °C and the gradient programme was as follows: 80% B decreasing to 50% B over 15 min, then to 5% B at 18 min until 21 min, increasing to 80% B at 24 min until 32 min. Total run time was 32 min with an injection volume of 10 μL. A mass spectrometer was operated in full scan mode with positive and negative polarity switching at 35k resolution at 200 *m/z*, with detection range of 85 to 1275 *m/z*, AGC target was 1e^6^ ions with a maximum injection time of 50 ms. Electro-spray ionisation source (HESI) was set to 4.0 kV voltage for positive and negative mode, and sheath gas was set to 50, aux gas to 20 and sweep gas to 2 arbitrary units, capillary temperature to 300 °C and probe heater temperature to 120 °C. The samples were analysed as a single batch to avoid batch-to-batch variation and randomised to account for LCMS system drift over time. Repeated analysis of pooled quality control samples was performed throughout the batch to confirm signal reproducibility.

### Data processing using IDEOM

The acquired LCMS data was processed in untargeted fashion using open source software, IDEOM [90] (http://mzmatch.sourceforge.net/ideom.php). Initially, *ProteoWizard* was used to convert raw LC-MS files to *mzXML* format and *XCMS* was used to pick peaks. *Mzmatch.R* was used to convert to *peakML* files, align samples and filter peaks using minimum detectable intensity of 100,000, relative standard deviation (RSD) of < 0.5 (reproducibility), and peak shape (codadw) of > 0.8. *Mzmatch* was also used to retrieve missing peaks and annotate related peaks. Default IDEOM parameters were used to eliminate unwanted noise and artefact peaks. Loss or gain of a proton was corrected in negative and positive ESI modes, respectively, followed by putative identification of metabolites by accurate mass within 3 ppm mass error searching against common metabolite databases including the Kyoto Encyclopedia of Genes and Genomes (KEGG), MetaCyc and LIPIDMAPS. To reduce the number of false positive identifications, retention time error was calculated for each putatively identified metabolite using IDEOM’s build-in retention time model which uses actual retention time data of authentic standards (~ 350 standards). Metabolites identified by comparison to authentic standards (including TCA cycle metabolites) are level 1 identifications according to the Metabolomics Standards Initiative, and all other putatively identified metabolites (including all peptides) are assigned as level 2. Statistical analysis on filtered data was performed using the Metaboanalyst web interface [91].

### Haemoglobin fractionation

The haemaglobin fractionation assay was adapted from [57]. Aliquots of 6.5 mL of 30–32 hr post invasion parasite cultures were adjusted to 8% parasitaemia and 2% haematocrit and then incubated with chloroquine, GSK-66, GSK-71 or ethanol (vehicle control) for 5 hrs. Treatments were performed in triplicate. Following incubation, the media was aspirated off and the culture was incubated with 2.3 mL of 0.1% saponin in 1× PBS with protease inhibitors (complete mini protease inhibitor cocktail (Roche)) for 10 min at 4 °C in order to lyse the iRBCs. The parasites were washed three times with PBS and stored at − 80 °C.

For the haemoglobin fractionation, lysed parasites were resuspended in 50 μL of Milli-Q water and sonicated for 5 min in a water bath sonicator. Following sonication, 50 μL of 0.2 M HEPES (pH 7.5) was added and the samples were centrifuged at 4000 rpm for 20 min. The supernatant containing the haemoglobin fraction was carefully transferred to new tubes and 50 μL of 4% of SDS was added before the samples were incubated at 95 °C for 5 min. Following heating, 50 μL of 0.3 M NaCl and 50 μL of 25% (v/v) pyridine (Sigma) in 0.2 M HEPES was added, the sample containing the haemoglobin fraction were vortexed and transferred to a 96-well plate.

The remaining pellets were treated with 50 μL of MilliQ water and 50 μL of 4% SDS and resuspended before being sonicated for 5 min and incubated at 95 °C for 5 min in order to solubilise the free haem. Following incubation, 50 μL of 0.2 M HEPES, 0.3 M NaCl and 25% pyridine were added to the samples. The samples were then subsequently centrifuged at 4000 rpm for 20 min. The supernatant was transferred to the 96-well plate, corresponding to the free haem fraction.

The remaining pellet containing the haemozoin fraction was solubilised by resuspending with 50 μL of MilliQ water and 50 μL of 0.3 M NaOH. The samples were sonicated for 15 min before 50 μL of 0.2 M HEPES, 0.3 M HCl and 25% pyridine was added. The samples were then transferred to the 96-well plate, corresponding to the haemozoin fraction. The total amount of haem in each fraction was quantified using a haem standard curve prepared from a 100 μg/mL standard solution of haematin in 0.3 M NaOH. Serial dilution of the standard curve was carried out in a 96-well plate in triplicate, and 50 μL of 0.2 M HEPES, 4% SDS, 0.3 M NaCl, 0.3 M HCl and 25% pyridine was added. The absorbance of the standard curve and each fraction was measured at a 405-nm wavelength using a Perkin Elmer Ensight Plate Reader. The samples were normalised via a paired analysis to the ethanol control and graphed as their fold change vs ethanol ± SEM. All fractions had > 2 replicates from 2 independent experiments.

## Supplementary information

**Additional file 1** : Table S1. Activities of azithromycin analogues.

**Additional file 2 **: Figure S1. Azithromycin analogues show improvement in invasion inhibitory activity. (A) Screening a panel of azithromycin analogues identified 7 with up to 6-fold lower invasion inhibitory IC_50_ activity in contrast to the parental azithromycin. IC_50_ Azithromycin 10 μM; GSK-4, 2.0 μM (Azithromycin vs GSK-4 *P* < 0.0001); GSK-5, 1.61 μM (Azithromycin vs GSK-5 P < 0.0001); GSK-56, 3.2 μM (Azithromycin vs GSK-56 P < 0.0001); GSK-8, 4.4 μM (Azithromycin vs GSK-8 *P* = 0.2); GSK-3, 1.8 μM (Azithromycin vs GSK-3 P < 0.0001); GSK-15, 3.6 μM (Azithromycin vs GSK-15 *P* < 0.001); GSK-72, 1.7 μM (Azithromycin vs GSK-72 P < 0.0001). Newly invaded ring-stage parasitemia was measured at 1 hr post invasion via flow cytometry. Data represents the mean of 2 (GSK 5) or more experiments expressed as percentage of non-inhibitory control. Error bars represent ± SEM. Dose response IC_50_s compared using extra sum of squares F-test. (B) The food-vacuole targeting antimalarial drugs chloroquine and quinine showed minimal invasion inhibitory activity at 10 μM while merozoite invasion was blocked by the invasion inhibitory control heparin (25 μg/mL). Data represents the mean of 3 experiments expressed as percentage of non-inhibitory control. Error bars represent ± SEM. Repeat measure data is available in Additional file 15 Supporting Value Data.

**Additional file 3** : Figure S2. Azithromycin analogues inhibit merozoite invasion irreversibly. Whether azithromycin analogues inhibited invasion through a direct effect on the merozoite, rather than an effect on the RBC, was assessed by directly treating and then washing the drug off purified merozoites. Analogue GSK-72 was chosen as a compound with improved invasion inhibitory activity over azithromycin with merozoites treated at 10 μM. The actin inhibitor cytochalasin D (cytoD, 500 μM) was included as an irreversible washout control. The RON2 binding peptide R1 (100 μg/mL) was included as a reversible control. Ring-stage parasitaemia of newly invaded parasites was determined ~ 30 min post invasion by flow cytometry, with results presented as % parasitaemia relative to a media control. Results represent the mean of 2 experiments and the error bars represent the ± SEM. Repeat measure data is available in Additional file 15 Supporting Value Data.

**Additional file 4 **: Figure S3. Growth inhibition profiles of azithromycin and analogues in parasites lacking the apicoplast. Early ring-stage *P. falciparum* parasites (0–4 hrs post-invasion) were treated with doubling dilutions of azithromycin and inhibition of growth measured for (A) 2 cycle (delayed death, 120 hr) assays (D10-PfPHG^apicoplast-null^ IC_50_, 4.5 μM; D10-PfPHG^wildtype^ IC_50_, 0.07 μM. P = < 0.0001) or (B) 44 hr (in-cycle) (D10-PfPHG^apicoplast-null^ IC_50_, 16 μM; D10-PfPHG^wildtype^ IC_50_, 11.3 μM. *P* = 0.24) assays. Parasitemia was measured at 120 hrs or 44 hrs post invasion, respectively, at schizont stage via flow cytometry. Data represents the mean of 3 (or more) experiments expressed as percentage of non-inhibitory control and error bars represent ± SEM. (C) There was no difference in 44 hr IC_50_s between D10-PfPHG^apicoplast-null^ and D10-PfPHG^wildtype^ parasites when treated with the azithromycin analogues GSK 1 (D10-PfPHG^apicoplast-null^ IC_50_, 0.028 μM; D10-PfPHG^wildtype^ IC_50_, 0.023 μM. *P* = 0.36) and GSK 66 (D10-PfPHG^apicoplast-null^ IC_50_, 0.009 μM; D10-PfPHG^wildtype^ IC_50_, 0.007 μM. *P* = 0.08). Data represents the mean of 2 (D10-PfPHG^apicoplast-null^) or 3 (D10-PfPHG^wildtype^) experiments expressed as percentage of non-inhibitory control and error bars represent ± SEM. Dose response IC_50_s compared using extra sum of squares F-test. Repeat measure data is available in Additional file 15 Supporting Value Data.

**Additional file 5** : Table S2. Azithromycin analogue activity across different age ranges of D10-PfPHG blood stage development.

**Additional file 6** : Table S3. Azithromycin analogue inhibition for chloroquine sensitive and resistant lines.

**Additional file 7 **: Table S4. Azithromycin analogue activity against *P. falciparum* D10-PfPHG and *P. knowlesi* YH1 parasites.

**Additional file 8 **: Table S5. Azithromycin analogue activity against the bacterial pathogen *Streptococcus pneumoniae* compared to *P. falciparum* D10-PfPHG.

**Additional file 9 **: Figure S4. Sparse partial least square-discriminant analysis (SPLS-DA) of *Plasmodium falciparum* (D10-PfPHG)-infected red blood cells following treatment with DHA (green), chloroquine (blue), azithromycin (light blue), GSK-5 (purple), GSK-71 (yellow), GSK-66 (grey), and ethanol control (red) from experiment 1. sPLS-DA showing scores plot for components one and two, the plots were generated using the top 10 metabolites for each component. Points represent individual sample replicates while the 95% confidence interval is represented by the shaded region. (File format .pdf).

**Additional file 10** : Table S6. Changes in metabolites upon azithromycin and analogue treatment shared with chloroquine treated parasites.

**Additional file 11** : Table S7. Changes in metabolites upon azithromycin and analogue treatment associated with the parasite TCA cycle.

**Additional file 12** : Table S8. Changes in metabolites upon azithromycin and analogue treatment mapping to haemoglobin after drug treatment.

**Additional file 13 **: Figure S5. Model for TCA metabolism following treatment of *Plasmodium falciparum* (D10-PfPHG)-infected red blood cells. Relative abundance of the TCA metabolites from infected red blood cells treated with DHA (blue), chloroquine (red), azithromycin (green), GSK-5 (purple), GSK-71 (orange), GSK-66 (black), compared with the Ethanol control from experiment 1. Data are represented as mean fold change from triplicate treatments multiplied by corresponding RSD values. Abbreviations: OAA, oxaloacetate; PEP, phosphoenolpyruvate.

**Additional file 14 **: Figure S6. Azithromycin does not pre-sensitise early-ring stages to chloroquine. Early ring-stage *P. falciparum* parasites (0–4 hrs post-invasion) were treated with doubling dilutions of chloroquine (IC_50_; 0–6 hrs, 0.73 μM), chloroquine + IC_10_ of azithromycin (IC_50_; 0–6 hrs, 1.1 μM) or highly potent analogue GSK-66 which features a chloroquinoline moiety (IC_50_; 0–6 hrs, 0.004 μM) for 0–6 hrs, prior to removal of drugs by washing. Comparison of the resulting in-cycle growth shows a small change between growth of chloroquine vs chloroquine + azithromycin treated parasites (*P* = 0.0041). This compares to a large difference between growth inhibitory IC_50_ of GSK-66 and chloroquine (*P* < 0.0001) and chloroquine + azithromycin (P < 0.0001), indicating that azithromycin does not potentiate ring stage activity of chloroquine. Parasitemia was measured at 44 hrs post invasion at schizont stage via flow cytometry. Data represents the mean of 3 (or more) experiments expressed as percentage of non-inhibitory control and error bars represent ± SEM. Dose response IC_50_s compared using extra sum of squares F-test. Repeat measure data is available in Additional file 15 Supporting Value Data. (File format .pdf).

**Additional file 15** : Supporting data values. Excel Spreadsheet containing repeat measure data for Figs. 3, 4, 5 and 7, and Additional files 2, 3, 4 and 15.

## Data Availability

All data generated or analysed during this study are included in this published article, its supplementary information files and publicly available repositories. The metabolomics spectrometry data and search results [92] supporting the conclusions of this article are available at the NIH Common Fund’s National Metabolomics Data Repository (NMDR) website, the Metabolomics Workbench, https://www.metabolomicsworkbench.org where it has been assigned Project ID (ST001315). The data can be accessed directly via it’s Project DOI: (10.21228/M8CX0M). This work is supported by NIH grant U2C-DK119886. Supporting data values for other experiments are included in Additional file 15 Supporting Data Values. Other datasets used and/or analysed during the current study are available from the corresponding author on request.

## Abbreviations

ACT: Artemisinin combination therapies
RBC: Red blood cell
Ca^2+^: Calcium
DMSO: Dimethyl sulfoxide
EtBr: Ethidium bromide
FIC: Fractional Inhibitory Concentration
FSC: Forward scatter
GFP: Green fluorescent protein
GSK: GlaxoSmithKline
HPLC: High-performance liquid chromatography
IC: Inhibitory concentration
IPP: Isoprenoid pyrophosphate
IPTp: Intermittent preventative treatment for malaria in pregnancy
KEGG: Kyoto Encyclopedia of Genes and Genomes
LC-MS: Liquid chromatography-mass spectrometry
MAPK: Mitogen-activated protein kinase
MIC: Minimum inhibitory concentration
*pf*: *Plasmodium falciparum*
*pk*: *Plasmodium knowlesi*
RSD: Relative standard deviation
RSA: Ring-stage survival assay
SEM: Standard error of the mean
WHO: World Health Organization
