# Supplementary material for: Retargeting azithromycin analogues to have dual-modality antimalarial activity
Source: BMC Biol. 2020 Sep 29;18:133. doi: 10.1186/s12915-020-00859-4 (PMC7526119; doi:10.1186/s12915-020-00859-4)
Supplement: Supplementary file 1 — Additional file 1 : Table S1. Activities of azithromycin analogues. [file 12915_2020_859_MOESM1_ESM.docx]

**Additional file 1: Table S1a.** Activities of N6-substituted analogues.





| **R^1^** | **R^2^** | **R^3^** | **Cmpnd #** | **Ref** | **D10-*Pf*PHG growth (% growth at 10 μM, *±SEM*)^a^** | **In-cycle (44 hr) growth D10-*Pf*PHG IC_50_ (μM, *±SEM*)^b^** | **Invasion inhibition (% rings at 1 μM, *±SEM*)^c^** | **D10-*Pf*PHG ^Apicoplast-null^**  **growth**  **(% growth at IC_90_ of D10-PfPHG WT, *±SEM*)^d^** | **In-cycle (24 hr) growth *Pk*YH1 IC_50_ (μM, *±SEM*)^e^** |
| --- | --- | --- | --- | --- | --- | --- | --- | --- | --- |
| desosaminyl | cladinosyl | Me | **AZR** | - | 65  *(1.5)* | 11.3  *(0.05)* | 106  *(7.1)* | 3.6  *(0.9)* | 16  *(1.8)* |
| desosaminyl | cladinosyl |  | **1** | ([1](#_ENREF_1)) | 10.4  *(1.9)* | 0.019  *(0.004)* | 95.5  *(6.8)* | 28.6  *(15)* | 0.2  *(0.005)* |
| desosaminyl | H |  | **2** | ([1](#_ENREF_1)) | 0.31  *(0.35)* | 0.024  *(0.004)* | 104  *(5.6)* |  |  |
| desosaminyl | cladinosyl |  | **3** | ([1](#_ENREF_1)) | 12  *(2.2)* | 0.18  *(0.03)* | 74  *(9.1)* | 5.0  *(3.9)* | 0.095  *(0.02)* |
| desosaminyl | cladinosyl |  | **4** | ([1](#_ENREF_1)) | 4  *(1.2)* | 0.2  *(0.01)* | 72  *(1.8)* |  |  |
| desosaminyl | cladinosyl |  | **5** | ([2](#_ENREF_2)) | 3  *(0.8)* | 0.2  *(0.01)* | 75  *(10.1)* | 0.3  *(0.1)* | 0.082  *(0.02)* |
| desosaminyl | cladinosyl |  | **6** | ([2](#_ENREF_2)) | 8  *(3.3)* | 0.28  *(0.05)* | 97  *(10)* | 1.8  *(0.9)* | 0.16  *(0.03)* |
| desosaminyl | H |  | **7** | ([1](#_ENREF_1)) | 13  *(3.6)* | 0.39  *(0.01)* | 99  *(17.6)* |  |  |
| desosaminyl | cladinosyl |  | **8** | ([1](#_ENREF_1)) | 0.97  *(3.1)* | 0.88  *(0.02)* | 76  *(11)* |  | 0.15  *(0.01)* |
| desosaminyl | cladinosyl |  | **9** | ([2](#_ENREF_2)) | 26  *(9.3)* | 0.44  *(0.07)* | 96  *(7.3)* | 4.4  *(3.7)* | 0.016  *(0.005)* |
| desosaminyl | cladinosyl |  | **10** | ([1](#_ENREF_1)) | 10 *(2)* | 0.48  *(0.04)* | 82  *(13.7)* | 2.2  *(1)* | 0.1  *(0.005)* |
| desosaminyl | cladinosyl |  | **11** | ([2](#_ENREF_2)) | 1.2  *(0.3)* | 0.53  *(0.08)* | 99  *(8.8)* |  |  |
| desosaminyl | cladinosyl |  | **12** | ([1](#_ENREF_1)) | 8  *(0.6)* | 0.59  *(0.08)* | 108  *(7.9)* |  |  |
| desosaminyl | H |  | **13** | ([1](#_ENREF_1)) | 13  *(3.6)* | 0.61  *(0.04)* | 100  *(7.8)* |  |  |
| desosaminyl | cladinosyl |  | **14** | ([3](#_ENREF_3)) | 3.1  *(2.5)* | 0.65  *(0.04)* | 93  *(3.7)* |  |  |
| desosaminyl | cladinosyl |  | **15** | ([1](#_ENREF_1)) | 1  *(0.3)* | 0.66  *(0.07)* | 87  *(4.5)* | 9.1  *(7.9)* |  |
| desosaminyl | cladinosyl |  | **16** | ([3](#_ENREF_3)) | 2  *(0.3)* | 0.7  *(0.04)* | 114  *(8)* |  |  |
| desosaminyl | cladinosyl |  | **17** | ([2](#_ENREF_2)) | 14  *(3.7)* | 0.7  *(0.05)* | 82  *(2.8)* | 7.3  *(5.5)* | 0.36  *(0.01)* |
| desosaminyl | cladinosyl |  | **18** | ([3](#_ENREF_3), [4](#_ENREF_4)) | 1  *(0.4)* | 0.84  *(0.08)* | 103  *(5.7)* |  |  |
| desosaminyl | cladinosyl |  | **19** | ([3](#_ENREF_3)) | 1  *(0.3)* | 0.88  *(0.08)* | 102  *(11.5)* |  |  |
| desosaminyl | cladinosyl |  | **20** | ([1](#_ENREF_1)) | 9  *(2.1)* | 1.13  *(0.16)* |  |  |  |
| desosaminyl | cladinosyl |  | **21** | ([2](#_ENREF_2)) | 22  *(6)* | 1.2  *(0.17)* |  |  |  |
| desosaminyl | cladinosyl |  | **22** | ([3](#_ENREF_3)) | 15  *(1.7)* | 1.22  *(0.01)* |  |  |  |
| desosaminyl | cladinosyl |  | **23** | ([2](#_ENREF_2)) | 0.33  *(0.2)* | 1.28  *(0.06)* |  |  |  |
| desosaminyl | cladinosyl |  | **24** | ([3](#_ENREF_3)) | 0.44  *(0.1)* | 1.3  *(0.1)* | 115  *(11)* |  |  |
| desosaminyl | cladinosyl |  | **25** | ([2](#_ENREF_2)) | 0.55  *(0.2)* | 1.4  *(0.1)* |  |  |  |
| desosaminyl | cladinosyl |  | **26** | ([3](#_ENREF_3), [4](#_ENREF_4)) | 0.08  *(0.05)* | 1.71  *(0.19)* |  |  |  |
| desosaminyl | cladinosyl |  | **27** | ([2](#_ENREF_2)) | 11  *(2)* | 1.84  *(0.1)* |  |  |  |
| desosaminyl | cladinosyl |  | **28** | ([1](#_ENREF_1)) | 2  *(0.5)* | 1.91  *(0.2)* |  |  |  |
| desosaminyl | cladinosyl |  | **29** | ([2](#_ENREF_2)) | 11  *(1.9)* | 2.2  *(0.4)* |  |  |  |
| desosaminyl | H |  | **30** | ([3](#_ENREF_3)) | 0.6  *(0.1)* | 2.32  *(0.2)* |  |  |  |
| desosaminyl | cladinosyl |  | **31** | ([2](#_ENREF_2)) | 0.16  *(0.08)* | 2.92  *(0.4)* |  |  |  |
| desosaminyl | cladinosyl |  | **32** | ([2](#_ENREF_2)) | 0.9  *(0.3)* | 3.07  *(0.3)* |  |  |  |
| desosaminyl | cladinosyl |  | **33** | ([1](#_ENREF_1)) | 5  *(1.4)* | 3.41  *(0.5)* |  |  |  |
| desosaminyl | cladinosyl |  | **34** | ([3](#_ENREF_3)) | 28  *(5)* | 4.2  *(0.2)* |  |  |  |
| desosaminyl | cladinosyl |  | **35** | ([3](#_ENREF_3)) | 0.54  *(0.1)* | 6.1  *(0.6)* |  |  |  |
| desosaminyl | cladinosyl |  | **36** | ([2](#_ENREF_2)) | 16  *(3.2)* | 6.5  *(0.6)* |  |  |  |
| desosaminyl | cladinosyl |  | **37** | ([1](#_ENREF_1)) | 6  *(1.7)* | 9.4  *(0.7)* |  |  |  |
| desosaminyl | cladinosyl |  | **38** | ([3](#_ENREF_3)) | 22  *(4.3)* | 23.5  *(0.7)* |  |  |  |
| desosaminyl | cladinosyl |  | **39** | ([2](#_ENREF_2)) | 98  *(3.5)* |  |  |  |  |
| H | H |  | **40** | ([1](#_ENREF_1)) | 98  *(2.8)* |  |  |  |  |
| H | H |  | **41** | ([1](#_ENREF_1)) | 102  *(2.2)* |  |  |  |  |
| desosaminyl | cladinosyl | H (des-*N*-methyl azithromycin) | **42** | - | 105  *(1.4)* |  |  |  |  |
| desosaminyl | cladinosyl | Me (azithromycin) | **43** | - | 112  *(2.7)* |  |  |  |  |
| desosaminyl | cladinosyl |  | **44** | ([1](#_ENREF_1)) | 105  *(2.5)* |  |  |  |  |
| desosaminyl | cladinosyl |  | **45** | ([2](#_ENREF_2)) | 99  *(3.3)* |  |  |  |  |
| desosaminyl | cladinosyl |  | **46** | ([3](#_ENREF_3)) | 83  *(2.3)* |  |  |  |  |
| desosaminyl | cladinosyl |  | **47** | ([2](#_ENREF_2)) | 64  *(4.8)* |  |  |  |  |
| desosaminyl | cladinosyl |  | **48** | ([1](#_ENREF_1)) | 97  *(1.8)* |  |  |  |  |
| desosaminyl | H |  | **49** | ([2](#_ENREF_2)) | 76  *(1.7)* |  |  |  |  |
| desosaminyl | cladinosyl |  | **50** | ([3](#_ENREF_3)) | 111  *(4.2)* |  |  |  |  |
| desosaminyl | cladinosyl |  | **51** | ([3](#_ENREF_3)) | 73.1  *(3.2)* |  |  |  |  |
| desosaminyl | cladinosyl |  | **52** | ([3](#_ENREF_3)) | 100  (4) |  |  |  |  |
| desosaminyl | cladinosyl |  | **53** | ([1](#_ENREF_1)) | 101  *(5.7)* |  |  |  |  |
| desosaminyl | cladinosyl |  | **54** | ([3](#_ENREF_3)) | 84.4  *(2.6)* |  |  |  |  |
| desosaminyl | H |  | **55** | ([1](#_ENREF_1)) | 98  *(3.3)* |  |  |  |  |

^a^ Drug treatment (10 μM) intracellular growth assay, from rings to late schizonts, with no rupture (*P. falciparum,* 0-44 hrs, n≥3).

^b^ Dilution series drug treatment intracellular growth assay, from rings to late schizonts, with no rupture (*P. falciparum,* 0-44 hrs. n≥3).

^c^ Drug treatment of merozoites (1 μM) for 10 mins prior to addition of RBCs. Parasitemia was measured by flow cytometry ~ 30 minutes post invasion (n=2 (for GSK 4, 5) or more).

^d^ Drug treatment of D10-PfPHG^apicoplast-null^ parasites at the concentration that inhibited D10-PfPHG WT parasite growth by 90% with 44 hr treatment (D10-PfPHG IC_90_). Intracellular growth assay, from rings to late schizonts, with no rupture (*P. falciparum,* 0-44 hrs, n≥3).

^e^ Dilution series drug treatment intracellular growth assay, from rings to late schizonts, with no rupture (*P. knowlesi,* 0-24 hrs, n≥2).

**Table S1b.** Activities of desosaminyl *N*-substituted analogues

| **R^2^** | **R^4^** | **Cmpnd #** | **Ref** | **D10-*Pf*PHG growth (% growth at 10 μM, *±SEM*)^a^** | **In-cycle (44 hr) growth D10-*Pf*PHG IC_50_ (μM, *±SEM*)^b^** | **Invasion inhibition (% rings at 1 μM, *±SEM*)^c^** | **D10-*Pf*PHG ^Apicoplast-null^**  **growth**  **(% growth at IC_90_ of D10-PfPHG WT, *±SEM*)^d^** | **In-cycle (24 hr) growth *Pk*YH1 IC_50_ (μM, *±SEM*)^e^** |
| --- | --- | --- | --- | --- | --- | --- | --- | --- |
| cladinosyl |  | **56** | ([5](#_ENREF_5)) | 6  *(1.5)* | 0.011 *(0.002)* | 77  *(3.2)* | 17  *(13.9)* | 0.031 *(0.008)* |
| H |  | **57** | ([5](#_ENREF_5)) | 7  *(1.1)* | 0.021 *(0.002)* | 94  *(5.3)* |  |  |
| cladinosyl |  | **58** | ([5](#_ENREF_5)) | 4  *(1.1)* | 0.048 *(0.003)* | 95  *(11)* | 1.6  *(0.9)* | 0.071  *(0.01)* |
| cladinosyl |  | **59** | ([5](#_ENREF_5)) | 8  *(1.3)* | 0.073 *(0.01)* | 108  *(8.2)* |  |  |
| cladinosyl |  | **60** | ([5](#_ENREF_5)) | 0.42  *(0.1)* | 0.098 *(0.01)* | 99  *(2.7)* |  |  |
| H |  | **61** | ([5](#_ENREF_5)) | 1  *(1)* | 0.5  *(0.04)* | 102  *(6)* |  |  |
| cladinosyl |  | **62** | ([5](#_ENREF_5)) | 0.3  *(0.1)* | 3.51 *(0.3)* |  |  |  |
| cladinosyl |  | **63** | ([5](#_ENREF_5)) | 12  *(2.2)* | 3.7  *(0.3)* |  |  |  |
| cladinosyl |  | **64** | ([5](#_ENREF_5)) | 101  *(2.1)* |  |  |  |  |
| H |  | **65** | ([5](#_ENREF_5)) | 101  *(2.5)* |  |  |  |  |

^a^ Drug treatment (10 μM) intracellular growth assay, from rings to late schizonts, with no rupture (*P. falciparum,* 0-44 hrs, n≥3).

^b^ Dilution series drug treatment intracellular growth assay, from rings to late schizonts, with no rupture (*P. falciparum,* 0-44 hrs. n≥3).

^c^ Drug treatment of merozoites (1 μM) for 10 mins prior to addition of RBCs. Parasitemia was measured by flow cytometry ~ 30 minutes post invasion (n=2 (for GSK 4, 5) or more).

^d^ Drug treatment of D10-PfPHG^apicoplast-null^ parasites at the concentration that inhibited D10-PfPHG WT parasite growth by 90% with 44 hr treatment (D10-PfPHG IC_90_). Intracellular growth assay, from rings to late schizonts, with no rupture (*P. falciparum,* 0-44 hrs, n≥3).

^e^ Dilution series drug treatment intracellular growth assay, from rings to late schizonts, with no rupture (*P. knowlesi,* 0-24 hrs, n≥2).

**Table S1c.** Activities of desosaminyl *O*-substituted analogues.

| **R^2^** | **R^5^** | **Cmpnd #** | **Ref** | **D10-*Pf*PHG growth (% growth at 10 μM, *±SEM*)^a^** | **In-cycle (44 hr) growth D10-*Pf*PHG IC_50_ (μM, *±SEM*)^b^** | **Invasion inhibition (% rings at 1 μM, *±SEM*)^c^** | **D10-*Pf*PHG ^Apicoplast-null^**  **growth**  **(% growth at IC_90_ of D10-PfPHG WT, *±SEM*)^d^** | **In-cycle (24 hr) growth *Pk*YH1 IC_50_ (μM, *±SEM*)^e^** |
| --- | --- | --- | --- | --- | --- | --- | --- | --- |
| cladinosyl |  | **66** | ([6](#_ENREF_6)) | 0.6  *(0.2)* | 0.007 *(0.001)* | 86  *(3.5)* | 12.4  *(8.2)* | 0.012 *(0.002)* |
| H |  | **67** | ([6](#_ENREF_6)) | 0.61  *(0.3)* | 0.024 *(0.004)* | 104  *(5.6)* |  |  |
| cladinosyl |  | **68** | ([6](#_ENREF_6)) | 8  *(1.5)* | 0.03 *(0.003)* | 95  *(12.3)* |  |  |
| cladinosyl |  | **69** | ([6](#_ENREF_6)) | 9  *(0.6)* | 0.03  *(0.04)* | 91  *(1.3)* |  |  |
| cladinosyl |  | **70** | ([6](#_ENREF_6)) | 12  *(1.4)* | 0.05 *(0.006)* | 79  *(3.1)* |  |  |
| cladinosyl |  | **71** | ([6](#_ENREF_6)) | 4  *(1.1)* | 0.053  *(0.005)* | 113  *(11)* | 9.7  *(5.7)* | 0.041  *(0.005)* |
| cladinosyl |  | **72** | - | 6  *(1.8)* | 0.27  *(0.1)* | 80  *(12.5)* | 1.6  *(1)* | 0.15  *(0.061)* |
| cladinosyl |  | **73** | ([6](#_ENREF_6)) | 0.9  *(0.3)* | 0.31  *(0.02)* | 96  *(11.4)* | 1.4  *(0.2)* | 0.248  *(0.07)* |
| cladinosyl |  | **74** | ([6](#_ENREF_6)) | 2  *(0.5)* | 0.34  *(0.03)* | 88  *(2.6)* |  |  |
| H |  | **75** | ([6](#_ENREF_6)) | 10  *(2.6)* | 0.35  *(0.03)* | 101  *(1.5)* |  |  |
| cladinosyl |  | **76** | ([6](#_ENREF_6)) | 9  *(1.4)* | 0.37  *(0.03)* | 90  *(7.7)* |  |  |
| H |  | **77** | ([6](#_ENREF_6)) | 1  *(0.3)* | 0.48  *(0.02)* | 99  *(4.4)* |  |  |
| cladinosyl |  | **78** | ([6](#_ENREF_6)) | 6  *(0.9)* | 0.51  *(0.04)* | 102  *(13.8)* |  |  |
| H |  | **79** | ([6](#_ENREF_6)) | 0.16  *(0.08)* | 1.2  *(0.01)* |  |  |  |
| H |  | **80** | ([6](#_ENREF_6)) | 8  *(1.6)* | 1.98  *(0.2)* |  |  |  |
| cladinosyl |  | **81** | ([6](#_ENREF_6)) | 10  *(2)* | 2.76  *(0.06)* |  |  |  |
| H |  | **82** | ([6](#_ENREF_6)) | 1  *(0.3)* | 4.8  *(0.1)* |  |  |  |
| cladinosyl |  | **83** | ([6](#_ENREF_6)) | 99  *(3)* |  |  |  |  |
| cladinosyl |  | **84** | ([6](#_ENREF_6)) | 102  *(3)* |  |  |  |  |

^a^ Drug treatment (10 μM) intracellular growth assay, from rings to late schizonts, with no rupture (*P. falciparum,* 0-44 hrs, n≥3).

^b^ Dilution series drug treatment intracellular growth assay, from rings to late schizonts, with no rupture (*P. falciparum,* 0-44 hrs. n≥3).

^c^ Drug treatment of merozoites (1 μM) for 10 mins prior to addition of RBCs. Parasitemia was measured by flow cytometry ~ 30 minutes post invasion (n=2 (for GSK 4, 5) or more).

^d^ Drug treatment of D10-PfPHG^apicoplast-null^ parasites at the concentration that inhibited D10-PfPHG WT parasite growth by 90% with 44 hr treatment (D10-PfPHG IC_90_). Intracellular growth assay, from rings to late schizonts, with no rupture (*P. falciparum,* 0-44 hrs, n≥3).

^e^ Dilution series drug treatment intracellular growth assay, from rings to late schizonts, with no rupture (*P. knowlesi,* 0-24 hrs, n≥2).

**References**

1. Peric M, Fajdetic A, Rupcic R, Alihodzic S, Ziher D, Bukvic Krajacic M, et al. Antimalarial activity of 9a-N substituted 15-membered azalides with improved *in vitro* and *in vivo* activity over azithromycin. J Med Chem. 2012;55(3):1389-401.

2. Hutinec A, Rupcic R, Ziher D, Smith KS, Milhous W, Ellis W, et al. An automated, polymer-assisted strategy for the preparation of urea and thiourea derivatives of 15-membered azalides as potential antimalarial chemotherapeutics. Bioorg Med Chem. 2011;19(5):1692-701.

3. Bukvic Krajacic M, Peric M, Smith KS, Schonfeld ZI, Ziher D, Fajdetic A, et al. Synthesis, structure-activity relationship, and antimalarial activity of ureas and thioureas of 15-membered azalides. J Med Chem. 2011;54(10):3595-605.

4. Bukvic Krajacic M, Novak P, Dumic M, Cindric M, Paljetak HC, Kujundzic N. Novel ureas and thioureas of 15-membered azalides with antibacterial activity against key respiratory pathogens. Eur J Med Chem. 2009;44(9):3459-70.

5. Starcevic K, Pesic D, Toplak A, Landek G, Alihodzic S, Herreros E, et al. Novel hybrid molecules based on 15-membered azalide as potential antimalarial agents. Eur J Med Chem. 2012;49:365-78.

6. Pesic D, Starcevic K, Toplak A, Herreros E, Vidal J, Almela MJ, et al. Design, synthesis, and *in vitro* activity of novel 2'-O-substituted 15-membered azalides. J Med Chem. 2012;55(7):3216-27.
