# Supplementary figures and images for: Retargeting azithromycin analogues to have dual-modality antimalarial activity
Source: BMC Biol. 2020 Sep 29;18:133. doi: 10.1186/s12915-020-00859-4 (PMC7526119; doi:10.1186/s12915-020-00859-4)

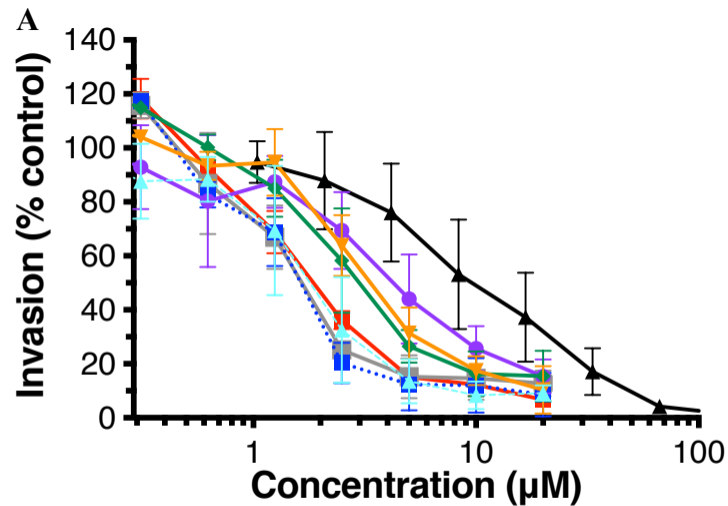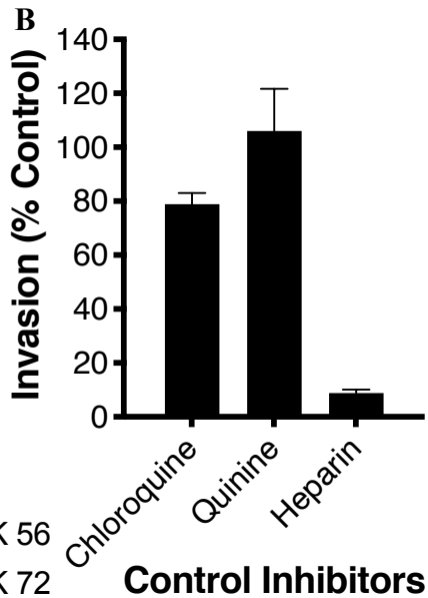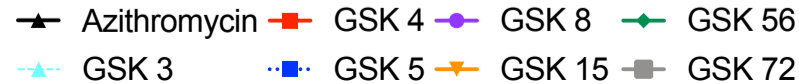

Supplement: Supplementary file 2 — Additional file 2 : Figure S1. Azithromycin analogues show improvement in invasion inhibitory activity. (A) Screening a panel of azithromycin analogues identified 7 with up to 6-fold lower invasion inhibitory IC50 activity in contrast to the parental azithromycin. IC50 Azithromycin 10 μM; GSK-4, 2.0 μM (Azithromycin vs GSK-4 P < 0.0001); GSK-5, 1.61 μM (Azithromycin vs GSK-5 P < 0.0001); GSK-56, 3.2 μM (Azithromycin vs GSK-56 P < 0.0001); GSK-8, 4.4 μM (Azithromycin vs GSK-8 P = 0.2); GSK-3, 1.8 μM (Azithromycin vs GSK-3 P < 0.0001); GSK-15, 3.6 μM (Azithromycin vs GSK-15 P < 0.001); GSK-72, 1.7 μM (Azithromycin vs GSK-72 P < 0.0001). Newly invaded ring-stage parasitemia was measured at 1 hr post invasion via flow cytometry. Data represents the mean of 2 (GSK 5) or more experiments expressed as percentage of non-inhibitory control. Error bars represent ± SEM. Dose response IC50s compared using extra sum of squares F-test. (B) The food-vacuole targeting antimalarial drugs chloroquine and quinine showed minimal invasion inhibitory activity at 10 μM while merozoite invasion was blocked by the invasion inhibitory control heparin (25 μg/mL). Data represents the mean of 3 experiments expressed as percentage of non-inhibitory control. Error bars represent ± SEM. Repeat measure data is available in Additional file 15 Supporting Value Data. [file 12915_2020_859_MOESM2_ESM.pdf]

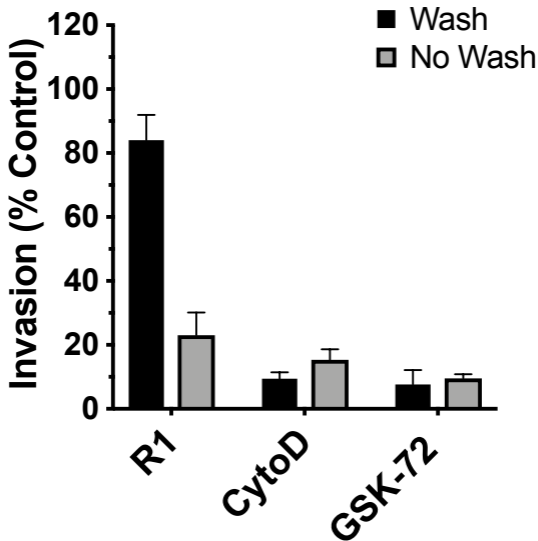

Supplement: Supplementary file 3 — Additional file 3 : Figure S2. Azithromycin analogues inhibit merozoite invasion irreversibly. Whether azithromycin analogues inhibited invasion through a direct effect on the merozoite, rather than an effect on the RBC, was assessed by directly treating and then washing the drug off purified merozoites. Analogue GSK-72 was chosen as a compound with improved invasion inhibitory activity over azithromycin with merozoites treated at 10 μM. The actin inhibitor cytochalasin D (cytoD, 500 μM) was included as an irreversible washout control. The RON2 binding peptide R1 (100 μg/mL) was included as a reversible control. Ring-stage parasitaemia of newly invaded parasites was determined ~ 30 min post invasion by flow cytometry, with results presented as % parasitaemia relative to a media control. Results represent the mean of 2 experiments and the error bars represent the ± SEM. Repeat measure data is available in Additional file 15 Supporting Value Data. [file 12915_2020_859_MOESM3_ESM.pdf]

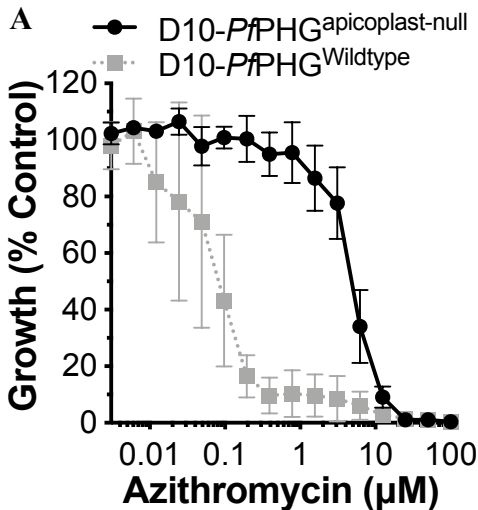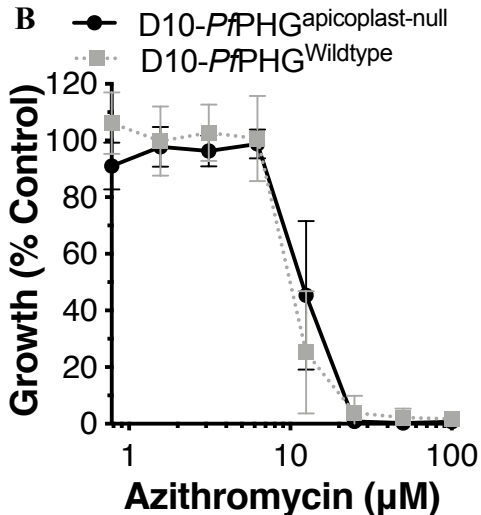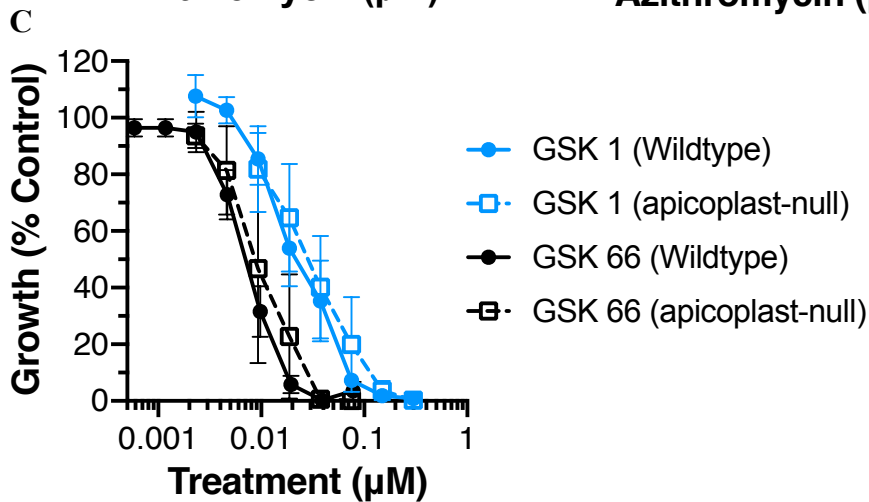

Supplement: Supplementary file 4 — Additional file 4 : Figure S3. Growth inhibition profiles of azithromycin and analogues in parasites lacking the apicoplast. Early ring-stage P. falciparum parasites (0–4 hrs post-invasion) were treated with doubling dilutions of azithromycin and inhibition of growth measured for (A) 2 cycle (delayed death, 120 hr) assays (D10-PfPHGapicoplast-null IC50, 4.5 μM; D10-PfPHGwildtype IC50, 0.07 μM. P = < 0.0001) or (B) 44 hr (in-cycle) (D10-PfPHGapicoplast-null IC50, 16 μM; D10-PfPHGwildtype IC50, 11.3 μM. P = 0.24) assays. Parasitemia was measured at 120 hrs or 44 hrs post invasion, respectively, at schizont stage via flow cytometry. Data represents the mean of 3 (or more) experiments expressed as percentage of non-inhibitory control and error bars represent ± SEM. (C) There was no difference in 44 hr IC50s between D10-PfPHGapicoplast-null and D10-PfPHGwildtype parasites when treated with the azithromycin analogues GSK 1 (D10-PfPHGapicoplast-null IC50, 0.028 μM; D10-PfPHGwildtype IC50, 0.023 μM. P = 0.36) and GSK 66 (D10-PfPHGapicoplast-null IC50, 0.009 μM; D10-PfPHGwildtype IC50, 0.007 μM. P = 0.08). Data represents the mean of 2 (D10-PfPHGapicoplast-null) or 3 (D10-PfPHGwildtype) experiments expressed as percentage of non-inhibitory control and error bars represent ± SEM. Dose response IC50s compared using extra sum of squares F-test. Repeat measure data is available in Additional file 15 Supporting Value Data. [file 12915_2020_859_MOESM4_ESM.pdf]

sPLSDA Scores Plot

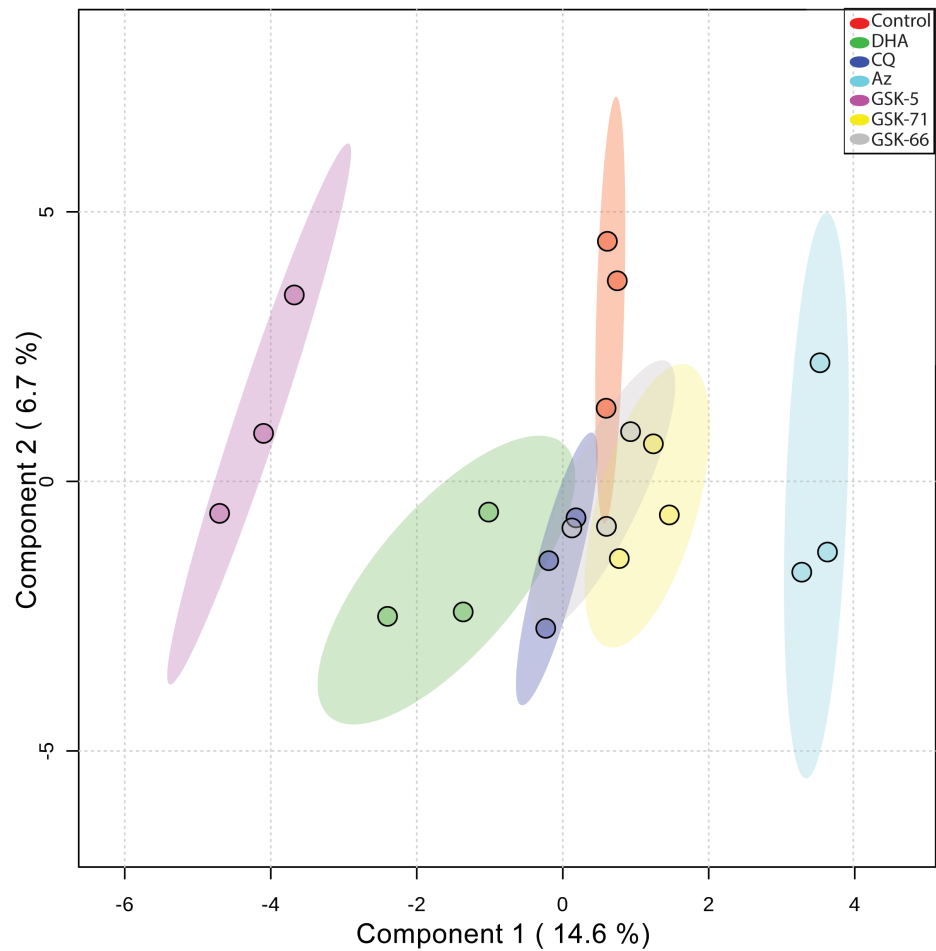

Supplement: Supplementary file 9 — Additional file 9 : Figure S4. Sparse partial least square-discriminant analysis (SPLS-DA) of Plasmodium falciparum (D10-PfPHG)-infected red blood cells following treatment with DHA (green), chloroquine (blue), azithromycin (light blue), GSK-5 (purple), GSK-71 (yellow), GSK-66 (grey), and ethanol control (red) from experiment 1. sPLS-DA showing scores plot for components one and two, the plots were generated using the top 10 metabolites for each component. Points represent individual sample replicates while the 95% confidence interval is represented by the shaded region. (File format .pdf). [file 12915_2020_859_MOESM9_ESM.pdf]

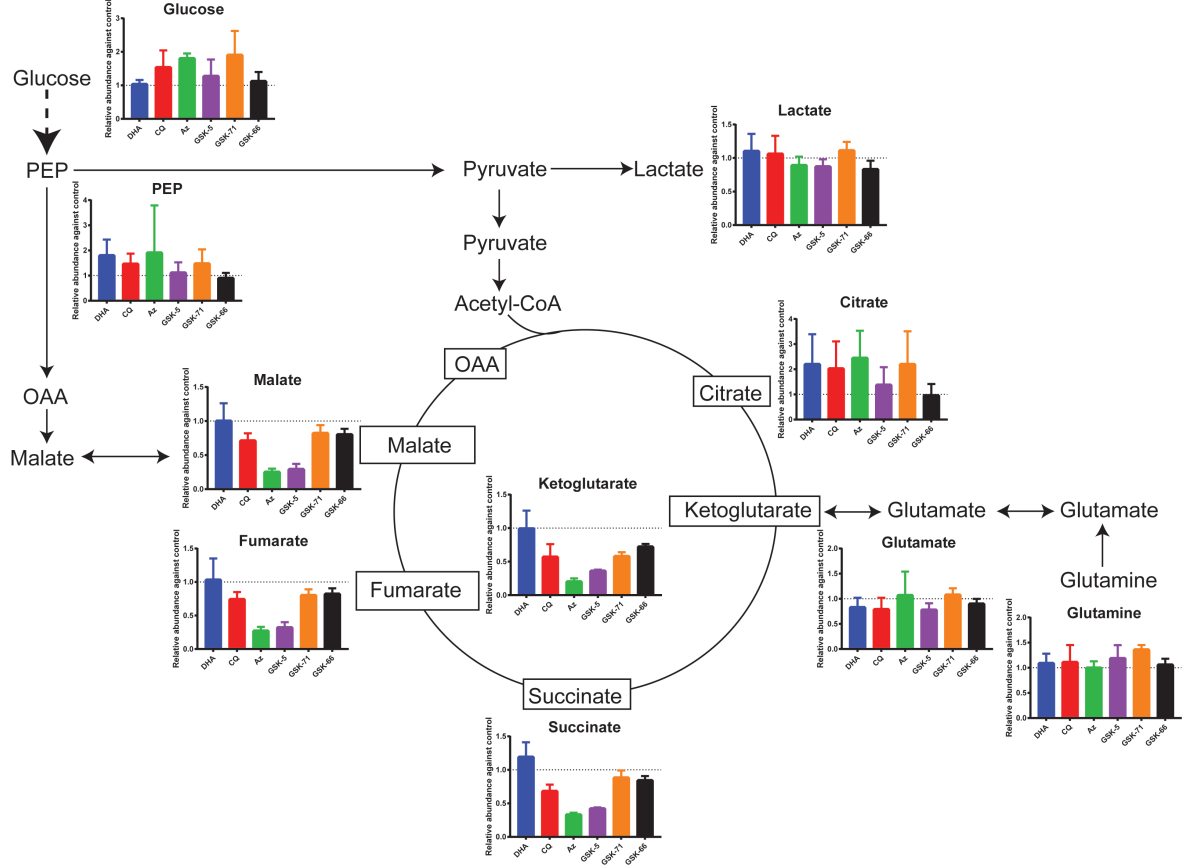

Supplement: Supplementary file 13 — Additional file 13 : Figure S5. Model for TCA metabolism following treatment of Plasmodium falciparum (D10-PfPHG)-infected red blood cells. Relative abundance of the TCA metabolites from infected red blood cells treated with DHA (blue), chloroquine (red), azithromycin (green), GSK-5 (purple), GSK-71 (orange), GSK-66 (black), compared with the Ethanol control from experiment 1. Data are represented as mean fold change from triplicate treatments multiplied by corresponding RSD values. Abbreviations: OAA, oxaloacetate; PEP, phosphoenolpyruvate. [file 12915_2020_859_MOESM13_ESM.pdf]

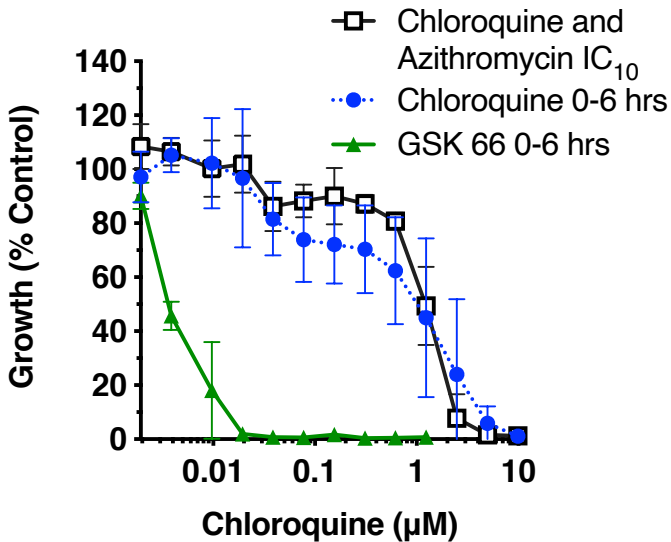

Supplement: Supplementary file 14 — Additional file 14 : Figure S6. Azithromycin does not pre-sensitise early-ring stages to chloroquine. Early ring-stage P. falciparum parasites (0–4 hrs post-invasion) were treated with doubling dilutions of chloroquine (IC50; 0–6 hrs, 0.73 μM), chloroquine + IC10 of azithromycin (IC50; 0–6 hrs, 1.1 μM) or highly potent analogue GSK-66 which features a chloroquinoline moiety (IC50; 0–6 hrs, 0.004 μM) for 0–6 hrs, prior to removal of drugs by washing. Comparison of the resulting in-cycle growth shows a small change between growth of chloroquine vs chloroquine + azithromycin treated parasites (P = 0.0041). This compares to a large difference between growth inhibitory IC50 of GSK-66 and chloroquine (P < 0.0001) and chloroquine + azithromycin (P < 0.0001), indicating that azithromycin does not potentiate ring stage activity of chloroquine. Parasitemia was measured at 44 hrs post invasion at schizont stage via flow cytometry. Data represents the mean of 3 (or more) experiments expressed as percentage of non-inhibitory control and error bars represent ± SEM. Dose response IC50s compared using extra sum of squares F-test. Repeat measure data is available in Additional file 15 Supporting Value Data. (File format .pdf). [file 12915_2020_859_MOESM14_ESM.pdf]
