# Supplementary material for: Retargeting azithromycin analogues to have dual-modality antimalarial activity
Source: BMC Biol. 2020 Sep 29;18:133. doi: 10.1186/s12915-020-00859-4 (PMC7526119; doi:10.1186/s12915-020-00859-4)
Supplement: Supplementary file 5 — Additional file 5 : Table S2. Azithromycin analogue activity across different age ranges of D10-PfPHG blood stage development. [file 12915_2020_859_MOESM5_ESM.docx]

| **Modification** | **Drug** | **IC_50_ (**μM**) 0-6 hrs PI** | **IC_50_ (**μM**) 0-12 hrs PI** | **IC_50_ (**μM**) 0-44 hrs PI** |
| --- | --- | --- | --- | --- |
|  | Azithromycin | 30 *(4.8)* | 16 *(3.7)* | 14 *(0.9)* |
|  | Chloroquine | 0.73 *(4.6)* | 0.15 *(0.02)* | 0.052 *(0.01)* |
|  | DHA | 0.011 *(0.001)* | 0.009 *(0.001)* | 0.008 *(0.001)* |
| Chloroquinoline | 66 | 0.004 *(0.001)* | 0.003 *(0.001)* | 0.003 *(0.001)* |
| Quinoline | 71 | 0.13 *(0.01)* | 0.11 *(0.01)* | 0.11 *(0.02)* |
| Naphthalene | 3 | 0.22 *(0.03)* | 0.15 *(0.04)* | 0.13 *(0.02)* |
| Substituted phenyl | 5 | 0.3 *(0.08)* | 0.37 *(0.05)* | 0.26 *(0.03)* |

**Additional file 5: Table S2. Azithromycin analogue activity across different age ranges of D10-PfPHG blood stage development**

Growth inhibitory IC_50_ values with different treatment durations for *P. falciparum* chloroquine sensitive D10-*Pf*PHG (Data from Figure 2D and 3A). Parasitaemia was measured by flow cytometry when parasites were around 44 hrs post-invasion. n ≥ 3.

PI= Post merozoite invasion.
