# Supplementary material for: Retargeting azithromycin analogues to have dual-modality antimalarial activity
Source: BMC Biol. 2020 Sep 29;18:133. doi: 10.1186/s12915-020-00859-4 (PMC7526119; doi:10.1186/s12915-020-00859-4)
Supplement: Supplementary file 6 — Additional file 6 : Table S3. Azithromycin analogue inhibition for chloroquine sensitive and resistant lines. [file 12915_2020_859_MOESM6_ESM.docx]

| **Modification** | **Drug** | **In-cycle (44 hr) growth D10-*Pf*PHG IC_50_ (μM, *±SEM*)^a^** | **In-cycle (44 hr) growth DD2 IC_50_ (μM, *±SEM*) ^a^** | **Fold change of In-cycle growth DD2 vs D10-*Pf*PHG IC_50_^b^** |
| --- | --- | --- | --- | --- |
|  | Azithromycin | 11.31 *(0.49)* | 15.6 *(2.1)* | 1.4 |
|  | Chloroquine | 0.052 *(0.006)* | 0.311 *(0.31)* | 5.9 |
| Chloroquinoline | 1 | 0.019 *(0.004)* | 0.082 *(0.02)* | 4.3 |
|  | 56 | 0.011 *(0.02)* | 0.093 *(0.04)* | 8.4 |
|  | 59 | 0.073 *(0.02)* | 0.049 *(0.005)* | 0.67 |
|  | 66 | 0.007 *(0.001)* | 0.043 *(0.002)* | 6.14 |
|  | 72 | 0.27 *(0.01)* | 0.065 *(0.004)* | 0.24 |
| Quinoline | 8 | 0.41 *(0.02)* | 0.52 *(0.1)* | 1.3 |
|  | 10 | 0.48 *(0.04)* | 0.748 *(0.1)* | 1.5 |
|  | 58 | 0.048 *(0.004)* | 0.056 *(0.01)* | 1.1 |
|  | 71 | 0.053 *(0.005)* | 0.16 *(0.02)* | 3 |
|  | 73 | 0.31 *(0.02)* | 0.48 *(0.2)* | 1.5 |
| Naphthalene | 3 | 0.183 *(0.02)* | 0.32 *(0.07)* | 1.7 |
|  | 15 | 0.67 *(0.07)* | 0.4 *(0.1)* | 0.6 |
| Substituted phenyl | 5 | 0.2 *(0.01)* | 0.4 *(0.05)* | 2 |
|  | 6 | 0.28 *(0.05)* | 0.27 *(0.07)* | 0.96 |
|  | 9 | 0.44 *(0.07)* | 0.24 *(0.04)* | 0.45 |
|  | 17 | 0.7 *(0.05)* | 0.54 *(0.06)* | 0.77 |

**Additional file 6: Table S3. Azithromycin analogue inhibition for chloroquine sensitive and resistant lines**

^a^ IC_50_ curves were performed for *P. falciparum* chloroquine sensitive D10-*Pf*PHG and chloroquine resistant DD2. Drug treatment from rings to late schizonts, with no rupture (*P. falciparum,* 0-44 hrs; *Pf*PHG and DD2), and parasitaemia measured by flow cytometry.

^b^ The fold change of IC_50_ values of lead analogues for D10-*Pf*PHG vs DD2 is indicated.

Results expressed as a percentage of non-inhibitory control (n ≥ 3).
