# Supplementary material for: Retargeting azithromycin analogues to have dual-modality antimalarial activity
Source: BMC Biol. 2020 Sep 29;18:133. doi: 10.1186/s12915-020-00859-4 (PMC7526119; doi:10.1186/s12915-020-00859-4)
Supplement: Supplementary file 7 — Additional file 7 : Table S4. Azithromycin analogue activity against P. falciparum D10-PfPHG and P. knowlesi YH1 parasites. [file 12915_2020_859_MOESM7_ESM.docx]

| **Modification** | **Compound** | **In-cycle (44 hr) growth D10-*Pf*PHG IC_50_ (μM, *±SEM*)^a^** | **In-cycle (24 hr) growth *Pk*YH1 IC_50_ (μM, *±SEM*)^a^** | **Fold change of Intracellular growth D10-*Pf*PHG vs *Pk*YH1 IC_50_** |
| --- | --- | --- | --- | --- |
|  | Azithromycin | 11.31 *(0.49)* | 16 *(1.8)* | 0.87 |
|  | Chloroquine | 0.052 *(0.006)* | 0.017 *(0.005)* | 3.1 |
|  | DHA | 0.00083 *(0.0001)* | 0.0024 *(0.001)* | 0.34 |
| Chloroquinoline | 1 | 0.019 *(0.004)* | 0.2 *(0.005)* | 0.095 |
|  | 56 | 0.011 *(0.02)* | 0.031 *(0.008)* | 0.35 |
|  | 66 | 0.007 *(0.001)* | 0.012 *(0.002)* | 0.58 |
|  | 72 | 0.27 *(0.01)* | 0.15 *(0.06)* | 0.18 |
| Quinoline | 8 | 0.41 *(0.02)* | 0.15 *(0.01)* | 2.7 |
|  | 10 | 0.48 *(0.04)* | 0.1 *(0.005)* | 4.8 |
|  | 58 | 0.048 *(0.004)* | 0.071 *(0.01)* | 0.68 |
|  | 71 | 0.053 *(0.005)* | 0.041 *(0.005)* | 1.29 |
|  | 73 | 0.31 *(0.02)* | 0.248 *(0.07)* | 1.25 |
| Naphthalene | 3 | 0.183 *(0.02)* | 0.095 *(0.02)* | 1.9 |
| Substituted phenyl | 5 | 0.2 *(0.01)* | 0.082 *(0.02)* | 2.4 |
|  | 6 | 0.28 *(0.05)* | 0.16 *(0.03)* | 1.75 |
|  | 9 | 0.44 *(0.07)* | 0.016 *(0.005)* | 33.1 |
|  | 17 | 0.7 *(0.05)* | 0.36 *(0.01)* | 1.9 |

**Additional file 7: Table S4. Azithromycin analogue activity against *P. falciparum* D10-PfPHG and *P. knowlesi* YH1 parasites**

^a^ IC_50_ curves were performed for *P. falciparum* D10-*Pf*PHG and *P. knowelsi* YH1. Drug treatment from rings to late schizonts, with no rupture (*P. falciparum,* 0-44 hrs; *P. knowlesi,* 0-24 hrs), and parasitaemia measured by flow cytometry.

^b^ The fold change of IC_50_’s of lead analogues for D10-*Pf*PHG vs *Pk*YH1 is indicated.

n = 2 (*P. knowlesi*), or n = 3 (D10-*Pf*PHG) experiments expressed as a percentage of non-inhibitory control.
