# Supplementary material for: Retargeting azithromycin analogues to have dual-modality antimalarial activity
Source: BMC Biol. 2020 Sep 29;18:133. doi: 10.1186/s12915-020-00859-4 (PMC7526119; doi:10.1186/s12915-020-00859-4)
Supplement: Supplementary file 8 — Additional file 8 : Table S5. Azithromycin analogue activity against the bacterial pathogen Streptococcus pneumoniae compared to P. falciparum D10-PfPHG. [file 12915_2020_859_MOESM8_ESM.docx]

| **Modification site** | **Compound** | ***S. pneumoniae* MIC average ^a^ (μM , *±SEM*)^a^** | **In-cycle (44 hr) growth D10-*Pf*PHG IC_50_ (μM, *±SEM*)^b^** | **Delayed-Death (120 hr) D10-*Pf*PHG IC_50_ (μM, *±SEM*)^c^** |
| --- | --- | --- | --- | --- |
|  | Azithromycin | 0.09 *(0.02)* | 11.3 *(0.49)* | 0.07 *(0.021)* |
| Desosaminyl *N*-  substituted | 57 | >10 | 0.021 *(0.002)* | 0.008 *(0.001)* |
|  | 66 | >10 | 0.007 *(0.001)* | ND |
|  | 71 | >10 | 0.053 *(0.005)* | 0.024 *(0.001)* |
|  | 78 | >10 | 0.51 *(0.04)* | ND |
| N6-substituted | 1 | 0.025 *(0.01)* | 0.019 *(0.004)* | ND |
|  | 4 | 0.035 *(0.01)* | 0.2 *(0.01)* | 0.013 *(0.04)* |
|  | 5 | 0.09 *(0.001)* | 0.2 *(0.01)* | 0.08 *(0.03)* |
|  | 6 | 0.157 *(0.02)* | 0.28 *(0.05)* | ND |
|  | 9 | 0.112 *(0.02)* | 0.44 *(0.07)* | ND |
|  | 11 | 0.075 *(0.003)* | 0.53 *(0.08)* | ND |
|  | 12 | 0.045 *(0.001)* | 0.59 *(0.08)* | 0.015 *(0.001)* |
|  | 16 | 0.023 *(0.001)* | 0.7 *(0.04)* | 0.07 *(0.004)* |
|  | 17 | 0.023 *(0.003)* | 0.7 *(0.05)* | ND |
|  | 21 | 0.112 *(0.02)* | 1.2 *(0.17)* | ND |
|  | 25 | 0.075 *(0.01)* | 1.4 *(0.1)* | ND |

**Additional file 8: Table S5. Azithromycin analogue activity against the bacterial pathogen *Streptococcus pneumoniae* compared to *P. falciparum* D10-PfPHG**

^a^Minimum inhibitory concentration (MIC) were determined as described in Methods. MIC dilution series were analyzed individually. n = 2, results expressed as a percentage of non-inhibitory control.

^b^Drug treatment of intracellular growth, from rings to late schizonts, with no rupture cycle (*P. falciparum,* 0-44 hrs; *Pf*PHG). n = 3.

^c^Drug treatment of delayed death, from rings to late schizonts, with two rupture cycle (*P. falciparum,* 0-120 hrs; *Pf*PHG). n = 2.

ND=Not Determined
