# Supplementary material for: Retargeting azithromycin analogues to have dual-modality antimalarial activity
Source: BMC Biol. 2020 Sep 29;18:133. doi: 10.1186/s12915-020-00859-4 (PMC7526119; doi:10.1186/s12915-020-00859-4)
Supplement: Supplementary file 10 — Additional file 10 : Table S6. Changes in metabolites upon azithromycin and analogue treatment shared with chloroquine treated parasites. [file 12915_2020_859_MOESM10_ESM.docx]

| **Putative metabolites** | **DHA** | **CQ** | **Az** | **GSK-5** | **GSK-71** | **GSK-66** | **Mapped to HB** | |
| --- | --- | --- | --- | --- | --- | --- | --- | --- |
|  |  |  |  |  |  |  | **Alpha subunit** | **Beta subunit** |
| Asn-His | 0.62 | 1.34 | **3.15** | **2.59** | **2.90** | 0.88 | GHG |  |
| Ala-Asp-Gly-Pro | 0.00 | 1.50 | **7.88** | **13.93** | **6.09** | 0.00 |  |  |
| Gln-Gln | 0.60 | **17.71** | **36.47** | **40.37** | **75.38** | 3.43 |  |  |
| Ala-Asn | 0.00 | 7.89 | **18.78** | **14.22** | **27.04** | 1.31 | GE |  |
| Thr-Arg | 0.69 | **3.83** | **8.74** | **5.92** | **12.99** | 1.50 |  |  |
| Pro-Ser | **0.62** | 2.15 | **3.75** | **3.56** | **6.47** | 0.91 | SP |  |
| Ala-Gly-Pro | 0.62 | **3.40** | **5.94** | **7.16** | **11.16** | 1.11 |  | PE |
| Val-Arg | 0.92 | **5.66** | **9.81** | **6.69** | **13.99** | 1.60 | RV |  |
| Ala-Arg | 0.90 | 18.29 | **11.33** | **13.97** | **26.58** | **2.36** |  |  |
| Pro-Tyr | **0.00** | **113.67** | **35.77** | **90.67** | **157.85** | **0.00** |  |  |
| Lys-Asn | **0.00** | **23.15** | **33.95** | **31.32** | **40.92** | **0.00** |  |  |
| Glu-Ile-Gln-Lys | **0.00** | **23.25** | **82.88** | **80.11** | **141.82** | **0.00** |  |  |
| Arg-Leu-Lys-Asp | **0.00** | **87.29** | **100.65** | **99.77** | **194.09** | **0.00** |  |  |
| Ala-Lys | **0.00** | **42.31** | **66.83** | **55.97** | **130.08** | **0.00** |  | KA |
| Lys-Tyr | **0.00** | **30.51** | **55.00** | **39.20** | **101.29** | **0.00** |  |  |
| Gly-Leu | 0.81 | **2.63** | **2.35** | **2.80** | **9.83** | 1.22 |  |  |
| Ala-Ala-Ser | 0.09 | 1.28 | **1.95** | 1.64 | 4.86 | 0.75 |  |  |
| Ile-Lys | 0.00 | 1.56 | **3.09** | **2.20** | **4.96** | 0.25 |  | LK |

**Additional file 10: Table S6a. Changes in metabolites upon azithromycin and analogue treatment shared with chloroquine treated parasites (Experiment 1)**

List of putative peptides that were significantly perturbed following treatment with DHA, chloroquine, azithromycin, GSK-5, GSK-71, and GSK-66 in three technical replicates. The order of the amino acids within the proposed peptide sequence has not been confirmed. The red shading denotes small peptides that were increased in abundance following treatment compared to Ethanol control, yellow denotes no change, and blue shading denotes peptides that were decreased. Values represent the fold-change relative to Ethanol control, and bold denotes changes that were statistically significantly different (t-test; p < 0.05; n = 3). The listed peptides (plus any isomeric peptides) were then investigated to determine whether it can be mapped to either the alpha or beta haemoglobin subunits.

| **Putative metabolites** | **DHA** | **CQ** | **Az** | **GSK-5** | **GSK-71** | **GSK-66** | **Mapped to HB** | |
| --- | --- | --- | --- | --- | --- | --- | --- | --- |
|  |  |  |  |  |  |  | **Alpha subunit** | **Beta subunit** |
| Asn-His | 1.19 | **6.35** | **3.90** | 2.10 | N/A | 1.47 | GHG |  |
| Ala-Asp-Gly-Pro | **0.00** | **0.00** | **13.75** | **40.09** | N/A | **0.00** |  |  |
| Gln-Gln | **0.00** | **315.03** | **255.49** | **108.08** | N/A | **32.39** |  |  |
| Ala-Asn | **4.25** | **52.56** | **37.06** | **25.54** | N/A | **4.11** | GE |  |
| Thr-Arg | 0.20 | **10.98** | **5.91** | 2.58 | N/A | 1.36 |  |  |
| Pro-Ser | 0.91 | **9.37** | **6.44** | **3.72** | N/A | 1.27 | SP |  |
| Ala-Gly-Pro | 0.91 | **9.67** | **7.43** | 4.13 | N/A | **1.40** |  | PE |
| Val-Arg | 0.53 | **9.50** | **6.30** | 2.60 | N/A | 1.20 | RV |  |
| Ala-Arg | 0.23 | **22.00** | 14.01 | 3.39 | N/A | 1.85 |  |  |
| Pro-Tyr | **0.00** | **117.98** | **68.16** | **25.73** | N/A | **0.00** |  |  |
| Lys-Asn | **2.65** | **86.06** | **74.45** | **27.25** | N/A | **7.84** |  |  |
| Glu-Ile-Gln-Lys | **0.00** | **67.38** | **61.65** | **11.74** | N/A | **0.00** |  |  |
| Arg-Leu-Lys-Asp | **0.00** | **225.30** | **108.47** | **44.30** | N/A | **0.00** |  |  |
| Ala-Lys | **0.00** | **78.30** | **59.52** | **14.22** | N/A | **0.00** |  | KA |
| Lys-Tyr | **0.00** | **63.25** | **32.47** | **5.08** | N/A | **0.00** |  |  |
| Gly-Leu | **1.44** | **6.78** | **3.57** | 2.54 | N/A | 1.40 |  |  |
| Ala-Ala-Ser | 1.03 | 7.99 | **6.48** | 3.05 | N/A | 1.19 |  |  |
| Ile-Lys | **0.00** | **167.67** | **118.47** | **44.37** | N/A | **22.38** |  | LK |

**Table S6b.** **Changes in metabolites upon azithromycin and analogue treatment shared with chloroquine treated parasites (Experiment 2)**

List of putative peptides that were identified in Experiment 1 as being significantly perturbed following treatment with DHA, chloroquine, azithromycin, GSK-5 and GSK-66 in three technical replicates. The order of the amino acids within the proposed peptide sequence has not been confirmed. The red shading denotes small peptides that were increased in abundance following treatment compared to Ethanol control, yellow denotes no change, and blue shading denotes peptides that were decreased. Values represent the fold-change relative to Ethanol control, and bold denotes changes that were statistically significantly different (t-test; p < 0.05; n = 3). The listed peptides (plus any isomeric peptides) were then investigated to determine whether it can be mapped to either the alpha or beta haemoglobin subunits. N/A= data not available.
