# Supplementary material for: Retargeting azithromycin analogues to have dual-modality antimalarial activity
Source: BMC Biol. 2020 Sep 29;18:133. doi: 10.1186/s12915-020-00859-4 (PMC7526119; doi:10.1186/s12915-020-00859-4)
Supplement: Supplementary file 11 — Additional file 11 : Table S7. Changes in metabolites upon azithromycin and analogue treatment associated with the parasite TCA cycle. [file 12915_2020_859_MOESM11_ESM.docx]

| **Metabolite** | **DHA** | **CQ** | **Az** | **GSK-5** | **GSK-71** | **GSK-66** |
| --- | --- | --- | --- | --- | --- | --- |
|  |  |  |  |  |  |  |
| Succinate* | 1.20 | **0.69** | **0.34** | **0.43** | 0.89 | **0.85** |
| 4-Aminobutanoate* | 0.86 | **0.63** | **0.14** | **0.25** | **0.64** | **0.75** |
| Ketoglutarate* | 1.00 | **0.58** | **0.21** | **0.37** | **0.59** | **0.73** |
| Malate* | 1.01 | **0.72** | **0.26** | **0.30** | 0.83 | 0.81 |
| Fumarate* | 1.04 | 0.75 | **0.28** | **0.33** | 0.81 | 0.83 |
| L-Pipecolate* | **0.49** | **0.53** | **0.07** | **0.09** | 0.81 | **0.58** |
| Asp-Gly | 0.90 | 0.85 | **0.27** | **0.28** | 0.91 | 0.97 |

**Additional file 11: Table S7a. Changes in metabolites upon azithromycin and analogue treatment associated with the parasite TCA cycle (Experiment 1)**

List of putative metabolites that were significantly perturbed following treatment with Az and GSK-5. The red shading denotes small peptides that were increased in abundance following treatment compared to Ethanol control, yellow denotes no change, and blue shading denotes peptides that were decreased. Values represent the fold-change relative to ethanol control, and bold denotes changes that were statistically significantly different (t-test; p < 0.05; n = 3).

* indicates metabolites that had identification confirmed using authentic standards (level 1).

| **Metabolite** | **DHA** | **CQ** | **Az** | **GSK-5** | **GSK-71** | **GSK-66** |
| --- | --- | --- | --- | --- | --- | --- |
|  |  |  |  |  |  |  |
| Succinate* | 1.23 | 0.88 | **0.64** | 1.33 | N/A | 1.24 |
| 4-Aminobutanoate* | 0.99 | **0.44** | **0.09** | 0.85 | N/A | 1.02 |
| Ketoglutarate* | 1.83 | 1.13 | 0.40 | 1.30 | N/A | 1.64 |
| Malate* | 1.18 | 0.95 | 0.50 | 1.49 | N/A | 1.26 |
| Fumarate* | 1.11 | 0.89 | 0.54 | 1.32 | N/A | 1.13 |
| L-Pipecolate* | 1.11 | 0.61 | 0.11 | 0.62 | N/A | 1.07 |
| Asp-Gly | 0.85 | 0.91 | **0.28** | 0.71 | N/A | 1.00 |

**Table S7b. Changes in metabolites upon azithromycin and analogue treatment associated with the parasite TCA cycle (Experiment 2).**

List of putative peptides that were identified in Experiment 1 as being significantly perturbed following treatment with Az and GSK-5. The red shading denotes small peptides that were increased in abundance following treatment compared to Ethanol control, yellow denotes no change, and blue shading denotes peptides that were decreased. Values represent the fold-change relative to ethanol control, and bold denotes changes that were statistically significantly different (t-test; p < 0.05; n = 3). N/A= data not available.

* indicates metabolites that had identification confirmed using authentic standards (level 1).
