# Supplementary material for: Retargeting azithromycin analogues to have dual-modality antimalarial activity
Source: BMC Biol. 2020 Sep 29;18:133. doi: 10.1186/s12915-020-00859-4 (PMC7526119; doi:10.1186/s12915-020-00859-4)
Supplement: Supplementary file 12 — Additional file 12 : Table S8. Changes in metabolites upon azithromycin and analogue treatment mapping to haemoglobin after drug treatment. [file 12915_2020_859_MOESM12_ESM.docx]

| **Peptide sequence** | **DHA** | **CQ** | **Az** | **GSK-5** | **GSK-71** | **GSK-66** | **Mapped to HB** | |
| --- | --- | --- | --- | --- | --- | --- | --- | --- |
|  |  |  |  |  |  |  | **Alpha subunit** | **Beta subunit** |
| Tyr-Arg | **0.40** | 0.69 | **1.48** | **0.30** | **0.90** | 0.98 | YR |  |
| Ala-Lys-Lys-Asp | 0.47 | **0.35** | 1.27 | 0.00 | 1.48 | 0.85 | SLDK |  |
| Asn-Leu-Pro-Pro | **0.37** | 0.64 | **1.90** | 0.00 | 1.69 | 0.98 |  | PPVQ |
| Ala-Asp-Gly-Tyr | **0.48** | 0.87 | 2.15 | 0.00 | 1.06 | 0.81 |  |  |
| Ala-Val-Gly-Pro | **0.54** | **0.55** | **0.58** | **0.03** | 0.84 | 0.82 |  |  |
| Ala-Glu-Glu-His | 0.75 | 1.01 | 1.14 | **0.09** | 1.19 | 0.94 | HVDD |  |
| Glu-Ala-Pro | **0.57** | **0.52** | 0.59 | 0.00 | **0.48** | 0.78 | PAE |  |
| Ala-Leu-Trp-Ser | **0.42** | 0.58 | 1.19 | 0.00 | 0.68 | 0.80 | PVNF |  |
| Trp-Asn | **0.44** | **0.59** | 0.82 | 0.00 | 0.52 | 0.98 |  | YH |
| Lys-Asp | 0.69 | **0.51** | 0.82 | **0.16** | **0.37** | 0.99 |  |  |
| Ala-His | **0.40** | **0.49** | **0.66** | **0.11** | **0.54** | 0.84 | AH |  |
| Glu-Glu-Gln-His | 0.00 | 1.06 | 0.00 | 0.00 | 0.76 | 0.61 |  |  |
| Asp-pro | **0.61** | 0.81 | **0.33** | **0.12** | 0.93 | 1.00 |  | VD |
| Lys-Pro | 0.82 | **0.58** | **0.56** | **0.29** | 0.98 | 0.99 |  | PK |
| Thr-Ala-Pro | **0.57** | **0.75** | **0.52** | **0.31** | 1.04 | 0.91 | TPA |  |
| Thr-Pro | **0.46** | **0.65** | **0.33** | **0.18** | 0.88 | 0.78 | PT |  |
| Val-Pro | **0.55** | **0.53** | **0.44** | **0.24** | **0.56** | **0.82** | PV |  |

**Additional file 12: Table S8a. Changes in metabolites upon azithromycin and analogue treatment mapping to haemoglobin after drug treatment (Experiment 1)**

List of putative peptides that were significantly perturbed following treatment with DHA, chloroquine, azithromycin, GSK-5, GSK-71 and GSK-66 in three technical replicates. The order of the amino acids within the proposed peptide sequence has not been confirmed. The red shading denotes small peptides that were increased in abundance following treatment compared to Ethanol control, yellow denotes no change, and blue shading denotes peptides that were decreased. Values represent the fold-change relative to Ethanol control, and bold denotes changes that were statistically significantly different (t-test; p < 0.05; n = 3).The listed peptides (plus any isomeric peptides) were then investigated to determine whether it can be mapped to either the alpha or beta haemoglobin subunits.

| **Peptide sequence** | **DHA** | **CQ** | **Az** | **GSK-5** | **GSK-71** | **GSK-66** | **Mapped to HB** | |
| --- | --- | --- | --- | --- | --- | --- | --- | --- |
|  |  |  |  |  |  |  | **Alpha subunit** | **Beta subunit** |
| Tyr-Arg | 0.78 | 1.17 | 0.70 | **0.42** | N/A | 1.08 | YR |  |
| Ala-Lys-Lys-Asp | 0.77 | 0.69 | 0.09 | 1.40 | N/A | 1.04 | SLDK |  |
| Asn-Leu-Pro-Pro | 0.80 | 0.88 | 0.81 | 0.80 | N/A | 1.31 |  | PPVQ |
| Ala-Asp-Gly-Tyr | **0.00** | **0.00** | **13.75** | **40.09** | N/A | **0.00** |  |  |
| Ala-Val-Gly-Pro | 1.23 | 0.76 | 0.07 | 0.69 | N/A | 1.55 |  |  |
| Ala-Glu-Glu-His | 0.81 | 1.20 | 0.18 | 0.48 | N/A | 0.75 | HVDD |  |
| Glu-Ala-Pro | 1.16 | **0.22** | 0.00 | 0.11 | N/A | 1.16 | PAE |  |
| Ala-Leu-Trp-Ser | 0.87 | 0.68 | 0.62 | 0.37 | N/A | 1.23 | PVNF |  |
| Trp-Asn | 0.99 | 0.66 | 0.09 | 0.36 | N/A | 1.20 |  | YH |
| Lys-Asp | 0.88 | 0.30 | 0.16 | 0.34 | N/A | 0.96 |  |  |
| Ala-His | 0.75 | **0.48** | **0.32** | **0.36** | N/A | 0.95 | AH |  |
| Glu-Glu-Gln-His | 0.00 | 1.93 | 0.00 | 0.00 | N/A | 0.69 |  |  |
| Asp-pro | 0.87 | 0.80 | 0.16 | 0.36 | N/A | 1.00 |  | VD |
| Lys-Pro | 0.87 | 0.88 | 0.33 | 0.60 | N/A | 0.95 |  | PK |
| Thr-Ala-Pro | 0.84 | 0.84 | 0.34 | 0.58 | N/A | 1.04 | TPA |  |
| Thr-Pro | 0.82 | 0.76 | 0.31 | 0.45 | N/A | 0.99 | PT |  |
| Val-Pro | 0.95 | 0.56 | 0.24 | 0.46 | N/A | 1.03 | PV |  |

**Table S8b. Changes in metabolites upon azithromycin and analogue treatment mapping to haemoglobin after drug treatment (Experiment2).**

List of putative peptides that were identified in Experiment 1 as being significantly perturbed following treatment with DHA, chloroquine, azithromycin, GSK-5, GSK-71 and GSK-66 in three technical replicates. The order of the amino acids within the proposed peptide sequence has not been confirmed. The red shading denotes small peptides that were increased in abundance following treatment compared to Ethanol control, yellow denotes no change, and blue shading denotes peptides that were decreased. Values represent the fold-change relative to Ethanol control, and bold denotes changes that were statistically significantly different (t-test; p < 0.05; n = 3). The listed peptides (plus any isomeric peptides) were then investigated to determine whether it can be mapped to either the alpha or beta haemoglobin subunits. N/A= data not available.
